# Polarising SNPs Without Outgroup

**DOI:** 10.1111/1755-0998.70105

**Published:** 2026-02-06

**Authors:** Jinyang Liang, Julien Y. Dutheil

**Affiliations:** ^1^ Department of Theoretical Biology Max Planck Institute for Evolutionary Biology Plön Germany

**Keywords:** ancestral recombination graph, outgroup, polarisation, unfolded site‐frequency spectrum

## Abstract

Asserting which allele is ancestral or derived, known as polarisation, is a prerequisite of many population and quantitative genetic methods. One important application is the inference of the unfolded site‐frequency spectrum (uSFS). The most widely used approaches are based on outgroup data. However, for studies on species with only distantly related outgroups, large divergence between the ingroup and outgroup can result in alignment difficulties and substantial missing data, causing many sites of interest to be lost. Here, we present PolarBEAR (Polarisation By Estimation of the Ancestral Recombination graph), a method that uses the local genealogies from the ancestral recombination graph (ARG) to infer ancestral states. We show that PolarBEAR reaches high accuracy in polarisation and uSFS estimation using simulations under several scenarios. This accuracy, however, heavily depends on the ARG reconstruction method employed. We also applied our method to human population data and compared it with the outgroup‐based method est‐sfs. Although PolarBEAR could not infer the ancestral state with high confidence at certain positions, it obtained results for positions that est‐sfs could not polarise due to missing outgroup data. The polarisation results of the two methods were highly consistent at positions inferred by both methods. The two methods inferred similar uSFS, with PolarBEAR estimating slightly fewer high‐frequency derived alleles. Furthermore, we demonstrate that PolarBEAR is robust across different mutation models in our simulations, while est‐sfs exhibits a bias in the presence of heterogeneous base composition. PolarBEAR can complement outgroup‐based methods, or replace them when no appropriate outgroup sequence is available.

## 1 Introduction

The process of inferring the ancestral vs. derived state of alleles at polymorphic sites is called “polarisation”. Polarised alleles are a required prior knowledge for many population genetics studies. Ancestral alleles are required as input to ARG inference methods such as Relate (Speidel et al. 2019) and tsinfer (Kelleher et al. 2019). This knowledge can also be used to detect adaptive evolution (Koenig et al. 2019), introgression (Fang et al. 2024), estimate allele age (Albers and McVean 2020; Pivirotto et al. 2024), and the strength of local selection (Stern et al. 2019). Furthermore, many methods have been developed to infer population genetic parameters from the distribution of the complete derived allele frequency spectrum, the so‐called unfolded site frequency spectrum (uSFS), calculated from polarisation results and containing signatures of demographic change (Keightley and Eyre‐Walker 2007; Gutenkunst et al. 2009), gene flow (Marchi and Excoffier 2020) and selection (Zeng et al. 2006; Keightley and Eyre‐Walker 2007; Schneider et al. 2011; Sendrowski and Bataillon 2024).

Ancestral or derived states cannot be directly observed from sequencing data. Current approaches typically rely on information from homologous sequences of closely related outgroup species. The simplest approach assumes the ancestral state is the state found in the outgroup (Voight et al. 2006; Schaefer et al. 2021), ignoring any evolutionary events on the outgroup branch since the common ancestor. Some early methods based on several outgroups and parsimony were proposed (Sabeti et al. 2007; Langley et al. 2012), but their limitations have long been recognised (Felsenstein 1981; Collins et al. 1994; Eyre‐Walker 1998). These methods tend to reconstruct common nucleotides as ancestral states, resulting in an overestimation of the number of rare changes. Furthermore, Hernandez, Williamson, Zhu, and Bustamante (2007) demonstrated that mis‐polarisation can bias downstream analyses: for example, they showed that polarisation errors can create the appearance of a fixation bias in favour of G and C alleles, which may be incorrectly attributed to positive selection or biased gene conversion. Similarly, Glémin et al. (2015) showed that even modest rates of polarisation errors can lead to misleading conclusions about the strength and direction of selection. Currently, the state‐of‐the‐art method for ancestral state inference is est‐sfs (Keightley et al. 2016; Keightley and Jackson 2018), based on maximum likelihood inference with up to three outgroups, which largely eliminates the bias compared to the parsimony method.

However, these outgroup‐based methods all have a common problem: due to species divergence, outgroup data are missing for some variants; this effect is even stronger when multiple outgroups are used. Hernandez, Williamson, and Bustamante (2007) used chimpanzee and rhesus macaque as outgroups to demonstrate the impact of this problem on the analysis of human population data. Reliance on outgroups makes the situation more difficult in the cases when (1) it is difficult to obtain suitable outgroup data for the focal species, for instance when it is a relict or non‐model species, and (2) it is more challenging to obtain homologous sequences with outgroups, such as in non‐conserved or rearranged genome regions.

Here we develop an outgroup‐free method, PolarBEAR (Polarisation By Estimation of the Ancestral Recombination graph), based on ARGs and an empirical Bayesian approach to infer ancestral states and estimate uSFS. Rather than relying on inter‐species relationships via outgroups, PolarBEAR uses within‐population genealogical information captured by ARGs. We assess the accuracy, power, and limits of this new method in comparison with classical approaches on simulated data and apply it to human population data from the 1000 Genomes Project as a test case.

## 2 Methods

### 2.1 Overview of PolarBEAR

**FIGURE 1:**
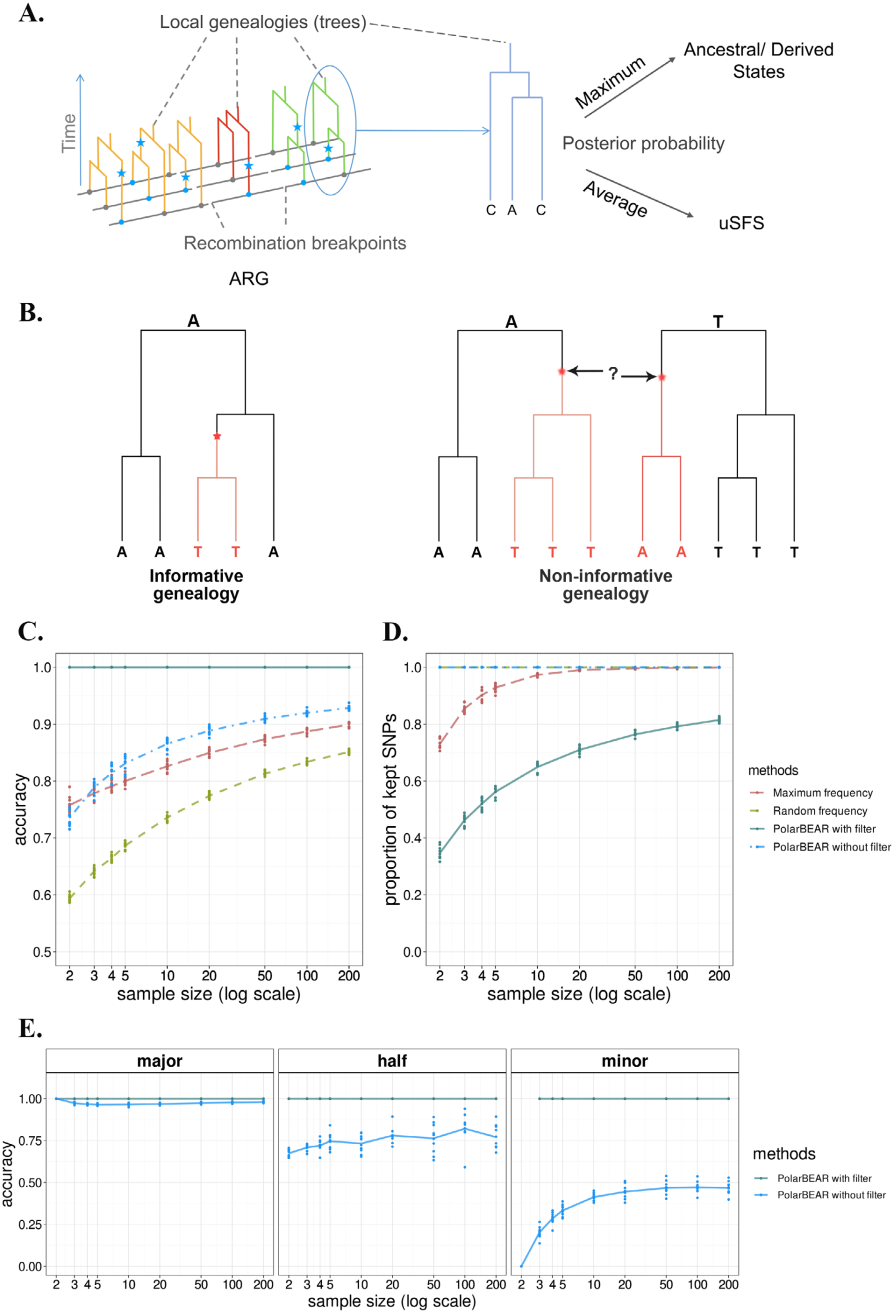
Polarising framework and performance of PolarBEAR with simulated ARG. (A) Inference framework of PolarBEAR. The left part shows an ARG, where genealogies of genomic segments separated by recombination breakpoints are represented in different colours; sites within the same segment share the same local genealogy, although the mutations (blue stars) occur at different positions. The local tree at one of the SNPs is circled as an example, illustrating how SNP‐specific genealogies are extracted from the ARG tree sequence. Posterior probabilities are then computed from the local tree and used for (i) maximum probability assignment of ancestral versus derived alleles, and (ii) posterior averaging of the uSFS. (B) Examples of informative and non‐informative genealogies. The red stars show the location of mutations, and the red branches represent lineages carrying the derived allele. (C and D) Comparison of accuracy, that is, the frequency of correctly inferred ancestral alleles (C) and proportion of analysed SNPs (D) between PolarBEAR with and without filtering of non‐informative genealogies, frequency‐based and random methods (colour) with different sample sizes. (E) Accuracy of PolarBEAR with and without filtering of non‐informative genealogies among the SNPs classified by their ancestral alleles as major, equal, and minor for different sample sizes. The points show 10 replicates in each group, and the lines represent their means.

The key idea of PolarBEAR is to use the information of genealogical relationships among sampled individuals within a population. This information is naturally captured by the ARG, a structure that connects a set of related genomes through a series of historical coalescence and recombination events (Griffiths and Marjoram 1997). Although ARGs are classically defined as graphs, a more practical and relevant view for our purposes is to treat them as a collection of local coalescent trees/genealogies that change along the genome and the recombination breakpoints that separate them (Minichiello and Durbin 2006; Wong et al. 2024). In this formulation, each non‐recombined segment of the genome shares a common genealogy describing the ancestral relationships among individuals. At any given position in the genome, a certain local genealogy can be independently extracted and analysed as a rooted tree with branch lengths representing the ages of the ancestral genotypes (Figure 1A).

Given the local genealogy, we infer the ancestral allele at the root of each tree using a model‐based ancestral state reconstruction procedure (Pseudocode S1) adapted from phylogenetics (Yang et al. 1995). For each possible ancestral allele at the root, we compute the joint likelihood of observing the genotypes at the leaf nodes under a given mutation model. This likelihood is calculated as the product of transition probabilities along all branches in the tree, where the transition probability depends on the mutation rate and the corresponding branch length. Using an empirical Bayesian approach, these likelihoods are computed for all possible root states and combined to compute the posterior probability of each nucleotide being the ancestral state. These posterior probabilities are used to derive the most likely ancestral allele for the site or to compute the posterior estimate of the uSFS, accounting for the uncertainty in the ancestral reconstruction.

However, inferring the ancestral state at the most recent common ancestor of the sample may be challenging. We introduce the term non‐informative genealogies (with respect to ancestral allelic reconstruction) to describe scenarios where the history of the sample provides little information for ancestral state reconstruction. For instance, when the history of the sample contains a single mutation, uncertainty happens when the mutation occurs on one of the branches at the root node (Figure 1B). The inferred ancestral state depends on whether the mutation occurs left or right of the root node, both scenarios being equally parsimonious. In terms of likelihood, the probability of each scenario only depends on the branch lengths and the chosen substitution model, resulting in very similar posterior probabilities. In contrast, informative genealogies arise when mutations occur on branches not directly connected to the root. When multiple mutations occur, more ambiguous scenarios can be observed.

### 2.2 Polarising from Local Genealogies

To determine the most likely ancestral allele, the posterior probabilities of assignments of the states were computed using the empirical Bayesian approach (Yang et al. 1995), and an equal‐rate model was used to compute transition probabilities. We performed a joint reconstruction (Yang et al. 1995) of all ancestral alleles at all inner nodes, as it does not require the averaging of conditional likelihoods at inner nodes, enabling the storage of log probabilities throughout the recursion and avoiding numerical underflow when the tree is large (Pseudocode S1).

Non‐informative genealogies, multiple mutations, and polytomies are determined based on the detection of mutations in the genealogies, which is achieved through parsimony implemented by the ‘Tree.map_mutations’ function from the tskit package. For each site, we considered all possible allelic states as candidate ancestral states. For each candidate, Tree.map_mutations returns the minimal set of mutations required to explain the observed genotypes. From these results, we used the following criteria:

Non‐informative genealogies—More than one candidate ancestral state produced the same minimal number of mutations; Multiple mutations—The minimum number of mutations inferred by parsimony among all scenarios with different candidate ancestral states is more than one; Polytomies with mutations—A mutation was placed on a node whose parent had more than two children.

### 2.3 Simulations

We used msprime (Baumdicker et al. 2022) to simulate ARG and genetic variation data.

#### 2.3.1 Default Simulation

We simulated one chromosome data with a length of 10 Mb, and with a constant population size of 30,000 diploid individuals (60,000 haplotypes), a mutation rate of 1.25e‐8 mutations per site per generation with the Jukes‐Cantor (JC) rate model (Jukes and Cantor 1969), and the deCODE recombination map (Halldorsson et al. 2019) of human chromosome 11 starting at position 20,004,256.

To test the impact of recombination rate, the sites were divided into high and low‐recombination‐rate categories, with the average recombination rate (9.69e‐09) as the boundary.

#### 2.3.2 Multiple Mutations

In one scenario, we generated simulations with a high mutation rate to obtain enough sites to test the impact of multiple mutation events. We used the same ARGs as the default simulations, discarding the original mutations and adding new mutations with a rate of 1e‐7 bp^−1^.

#### 2.3.3 Demographic Scenarios

We simulated two demographic change scenarios, population size increasing from 960 to 46,000 and decreasing from 46,000 to 960, respectively, with a logarithmic change within the last 150,000 generations.

#### 2.3.4 Unequal Transitions and Transversions

To assess the effect of multiple mutations, we removed the original, JC‐generated mutations from the ARGs and replaced them with new mutations generated under a Kimura two‐parameter mutation model (Kimura 1980) with the transition/transversion rate ratio κ=5.

#### 2.3.5 GC‐Biased Root Distribution

We simulated ingroup data with population parameters identical to the default simulations, but with a simulated sequence length of 50 Mb in order to have a sufficient number of sites after classifying them by mutation type. Two outgroup populations with the same size (kept constant) as the ingroup were added, with divergence times at 1.5e6 and 9e5 generations ago, respectively. In non‐constant demographic scenarios, changes in population size occur in the ingroup, and other parameters are identical to the default simulation scenario. The JC mutation model was used, with equal rates, but a GC‐to‐AT frequency ratio of 3:2 was used for the root distribution, while equilibrium frequencies remained equal to 0.25.

#### 2.3.6 Background Selection

We used tree sequence data generated by Barroso and Dutheil (2023), where forward simulations were performed with SLiM 3.00 (Haller and Messer 2019) using *Drosophila melanogaster* chromosome 2 L as a template. Briefly, a 23.51 Mb genome region was simulated, with recombination rates from (Comeron et al. 2012) in 100 kb windows and gene annotations from Ensembl release 103 (Cunningham et al. 2022). The deleterious mutations were modelled in exons, with fitness effects drawn from a negative gamma distribution (shape = 1.0, mean = −5/10,000). The population size was set to 10,000 diploid individuals (20,000 haplotypes), and simulations were run for 30,000 generations. The deleterious mutation rate was fixed at 1e‐7 per bp per generation, and recombination rates scaled by a factor of 10.

The simulations were saved as tree sequences, which were further processed by the python module ‘pyslim’. After recapitation of the ARG where needed, the integer‐coded SLiM mutations were converted into standard nucleotide states under the JC model and neutral mutations were added with msprime using a uniform mutation rate of 1e‐7 per bp.

### 2.4 Inference of ARGs

A wide range of sample sizes was used: 2, 3, 4, 5, 10, 20, 50, 100, and 200 diploid individuals, with ten replicates in each case. Small sample sizes are typical of datasets of non‐model organisms, while sample sizes in the hundreds are common for populations of Drosophila or Arabidopsis, for instance. Sample sizes in the thousands and beyond are available for Humans, but were not assessed here owing to the limitations of running time and memory; for similar reasons, the maximum sample size for PSMC was 10, while that of other methods was 100. The sample size 200 was only used for known ARGs.

The PSMC algorithm, as implemented in the iSMC program (Barroso et al. 2019), was used with 30 time intervals. The TMRCAs at each SNP position were computed as the posterior average over all 30 time intervals.

The gamma‐SMC (Schweiger and Durbin 2023) program was used with default parameters. The ratio of mutation to recombination rate was set to 0.78, which is the true ratio in the simulations and is similar to real human data.

The local TMRCAs of the positions with SNPs output by PSMC and gamma‐SMC were used to construct a distance matrix describing the haploid relationships. The “scipy.cluster.hierarchy.linkage” function (Virtanen et al. 2020) was used to implement UPGMA clustering to obtain the tree from the distance matrix.

The parameter ‐*t* was used in RENT+ (Mirzaei and Wu 2017) to get better branch length estimates.

In addition to the control for evaluating the effects of input ancestral states, the major alleles were treated as the ancestral alleles as the input of tsinfer (Kelleher et al. 2019), and if the two alleles each account for 50%, it was treated as missing data. After inferring the tree topology with tsinfer, tsdate (Wohns et al. 2022) was used to estimate branch lengths. For simulated data with demographic changes, the effective population size was calculated using $N_{e}=\theta /\left(4\mu \right)$ for the input of tsdate, where the scaled mutation rate θ was computed from the data using Watterson's estimator (Watterson 1975).

For PSMC, gamma‐SMC and RENT+, the local tree topologies were recorded only at SNP positions in the tskit tree sequence files. In order to meet tskit's requirements for node time, the times of non‐leaf nodes less than 1e‐12 were set to 1e‐12; if the time difference between a pair of internal parent and child nodes does not exceed 1e‐14, the child node is deleted, creating polytomies.

### 2.5 Real Data

#### 2.5.1 Data Preparation

The phased SNP data and the mask file for chromosome 1 were obtained from the 1000 Genomes Project's FTP server. Data specific to the African population, Mende, Sierra Leone (MSL), containing 87 diploid individuals, were selected for this study. All child samples in trios were excluded and the variants were filtered to include only SNPs that passed all filters. Multiple records of the same positions were collapsed into single multiallelic records. Regions of low confidence or known artefacts were excluded by applying the “StrictMask”.

The MultiZ 20‐species alignment file was downloaded from the UCSC Genome Browser. The VCF file including polymorphic and non‐polymorphic sites of Human, Orangutan, and Macaque was generated using MafFilter (Dutheil et al. 2014). The processed multi‐species alignment VCF file was merged with the cleaned population‐specific VCF file, keeping only positions present in both sets. Python scripts were used to process the merged VCF file and generate the est‐sfs input file.

#### 2.5.2 Annotation

The SNPs were annotated using the SnpEff version 5.2c (Cingolani et al. 2012). The reference genome used for annotation was GRCh38, with gene annotations from Ensembl release 99. The classic mode of SnpEff was used.

#### 2.5.3 uSFS Estimation of est‐sfs Using Partial SNPs

First, the posterior probabilities of the major allele as the ancestral state were obtained from the est‐sfs output. At the SNP i, if the output posterior probability $P_{\text{output}}$ > 0.5, the major allele is the ancestral state, and the posterior probability $P_{i}=P_{\text{output}}$; otherwise, the minor allele is the ancestral state, and the posterior probability $P_{i}=1-P_{\text{output}}$. Then, for the selected subset of SNPs, uSFS was estimated using the formula used for PolarBEAR in the section *Estimating the Unfolded Site Frequency Spectrum*.

## 3 Results and Discussion

We first assessed PolarBEAR's accuracy using simulated data across a range of scenarios. Subsequently, we applied PolarBEAR to human genomic data to compare it with the outgroup‐based method est‐sfs.

### 3.1 Accuracy of Allele Polarisation When the ARG Is Known

We first assessed the accuracy of the ancestral reconstruction in the ideal scenario where the true ARG is known and there is only one mutation per site (Figure 1C), using simulations (see Methods). We further demonstrate the impact of filtering out the non‐informative genealogies (Figure 1C). The proportion of accurately inferred ancestral states (hereby referred to as “accuracy”) increases with the sample size. The accuracy of PolarBEAR is generally higher than considering the Major allele as the ancestral one (hereby referred to as the “Maximum frequency” method) or sampling according to the allelic frequency (hereby referred to as the “Random frequency” method). We note that for sample sizes above 20 diploid individuals, the average accuracy of the two latter methods is virtually identical on average. Using PolarBEAR while filtering out the cases of non‐informative genealogies leads to an accuracy of 100% for all sample sizes, showing that when the ARG is known, the non‐informative topologies account for the total of the erroneous inference. The proportion of positions with non‐informative topologies is very high for small sample sizes (65% of the topologies for two diploids). It decreases as the sample size increases (20% of positions for 200 diploids, Figure 1D).

We then compare the inference with the frequency methods in more detail. By construction, the maximum frequency method will have an accuracy of 100% when the true ancestral allele is the major one and 0% when the true ancestral allele is the minor one. Using the true genealogy, PolarBEAR recovers the correct ancestral state with 100% accuracy when the ancestral allele is the major one, but the accuracy drops to 75% when the two alleles are in equal frequencies and to below 50% when the ancestral allele is the minor one (Figure 1E). In the latter case, the accuracy is even lower when the sample size is smaller than ten diploid individuals. When filtering out non‐informative genealogies, the accuracy becomes 100% in all three cases, demonstrating that erroneous inferences stem from non‐informative genealogies, which are more frequent when the ancestral allele is the minor one. The proportion of these genealogies increases with smaller sample sizes and reaches 100% for two diploids (the minor allele is in frequency ¼ and if it is the ancestral one, the corresponding individual is necessarily on a branch connected to the root). In subsequent results, we filter the non‐informative genealogies when polarising SNPs with the PolarBEAR method.

Finally, we assess the impact of multiple mutations on the ancestral state reconstruction (Figure S1). To obtain enough sites with multiple mutation events, simulations with a higher mutation rate of 1e‐7 bp^−1^ were employed (see Methods). PolarBEAR infers the ancestral allele with an accuracy of more than 90% in samples of more than ten individuals when more than two alleles are present (Figure S1A). This means that the assumption of an infinite site model is not necessary when the ARG is accurate. When we further exclude the genealogies with homoplasy (two alleles but multiple mutations), the accuracy reaches 100% (Figure S1A,C), indicating that the same mutation occurring in different branches (homoplasy) is the main reason for incorrect inference. The effect is stronger when the true ancestral allele is the minor one. Although the accuracy increases with sample size regardless of the ancestral allele frequency, it does not exceed 75% at a sample size of 20 when the ancestral allele is the non‐major one. In comparison, it is close to 100% when the ancestral is the major one for all the sample sizes (Figure S1C).

### 3.2 Accuracy When the ARG Is Inferred from the Data

We next examine how the method performs in practical cases when the ARG is unknown and must first be inferred from the data. Although some methods, such as ARGweaver (Rasmussen et al. 2014; Hubisz and Siepel 2020), can accommodate unphased data (not considered here due to prohibitive runtime at the genome‐wide scale), it should be noted that most ARG inference approaches, including those evaluated in this study, require phased data due to their principles. Several recent studies have compared the accuracy and applicability of different ARG methods (Y C Brandt et al. 2022; Brandt et al. 2024; Nielsen et al. 2025). It is difficult, however, to assess a priori which method performs best for polarisation because accuracy involves multiple aspects (recombination breakpoints, local topology, and branch lengths), and robustness depends on the task and underlying evolutionary scenario. ARGs can be directly obtained from methods such as tsinfer (Kelleher et al. 2019), Relate, ARG‐Needle (Zhang et al. 2023) and RENT+ (Mirzaei and Wu 2017); a brief overview of the most used ARG inference methods at the time of writing is summarised in Table 1. Alternatively, local genealogies can be reconstructed from the set of times to the most recent common ancestor (TMRCA) of every pair of genomes estimated using sequentially Markovian coalescent (SMC)‐based methods. For the latter, these methods rely mainly on heterozygosity patterns rather than on genotype similarity or allele polarisation, and are therefore relatively independent of the polarisation framework considered here. The output TMRCAs from these methods of all pairs of haplotypes in the sample at segregating sites are combined into distance matrices, which we then use to reconstruct the rooted local genealogies with the unweighted pair group method with arithmetic mean (UPGMA) algorithm, in a manner related to previous work (Zhang et al. 2023). We therefore compared four representative approaches: SMC‐based methods PSMC (Li and Durbin 2011) and the faster gamma‐SMC (Schweiger and Durbin 2023), which can be applied to larger samples; RENT+ as an even more scalable alternative; and tsinfer which requires ancestral states as input. We assessed whether accurate polarisation could still be achieved using ARGs obtained while considering major alleles as ancestral (possibly using an iterative approach that would lead to the joint inference of ARGs and ancestral alleles). As tsinfer only reconstructs the tree topology, tsdate (Wohns et al. 2022) is used next to determine branch lengths (hereby referred to as “tsinfer + tsdate”). We compare these different reconstruction methods and assess their impact on the ancestral allele reconstruction. For PSMC, the maximum sample size used was ten diploids due to computational resource limitations and 100 for the other methods.

**TABLE 1:** ARG inference methods.

Method	Framework	Additional required inputs	Advantages	Included in this study
ARGWeaver (Rasmussen et al. 2014)	SMC‐based	/	Accuracy	No
RENT+ (Mirzaei and Wu 2017)	Heuristics	/	Scalability (faster than ARGweaver)	Yes
SARGE (Shaw et al. 2004)	Heuristics	Ancestral states	Scalability (faster than ARGweaver)	No
ARG‐Needle (Zhang et al. 2023)	SMC‐based	Genetic map	Accuracy (among the more scalable methods like Relate and tsinfer)	No
Relate (Speidel et al. 2019)	L&S (Li and Stephens algorithm, Li and Stephens 2003)	Ancestral states	Scalability	No
Tsinfer + tsdate (Kelleher et al. 2019; Wohns et al. 2022).	L&S	Ancestral states; mutation rate	Scalability, speed	Yes

Unlike the true ARGs, the inferred ARGs inevitably contain uncertainty and topological errors, though the form of this uncertainty depends on the inference method. In the methods we evaluate here, such as RENT+ and tsinfer + tsdate, uncertainty is often represented by polytomies in the inferred topologies. When mutations occur at polytomies, we cannot rule out that the underlying true genealogies are non‐informative. However, topological errors may not affect polarisation, for instance, when they are under a common ancestor node without any mutations below it. In other cases, they may result in an unreasonably high number of inferred mutation events. We reconstructed the positions of mutations along the inferred genealogies and filtered out cases where mutations occurred under a polytomy or when multiple mutations were inferred. The first filtering scheme mainly affects RENT+ and tsinfer + tsdate, as mentioned above, and larger sample sizes generate more short internal branches, resulting in an increased number of unresolved branches represented as polytomies (Figure S2B). While the latter is useful for PSMC and gamma‐SMC, both of which have more noticeable effects when the true ancestral allele is the minor one or half‐half (Figure S2). When the sample size is two, all the genealogies in ARGs obtained by tsinfer are polytomies at the root node, so when the first filter is implemented, no analysable SNPs are kept (Figures S2 and 2B).

**FIGURE 2:**
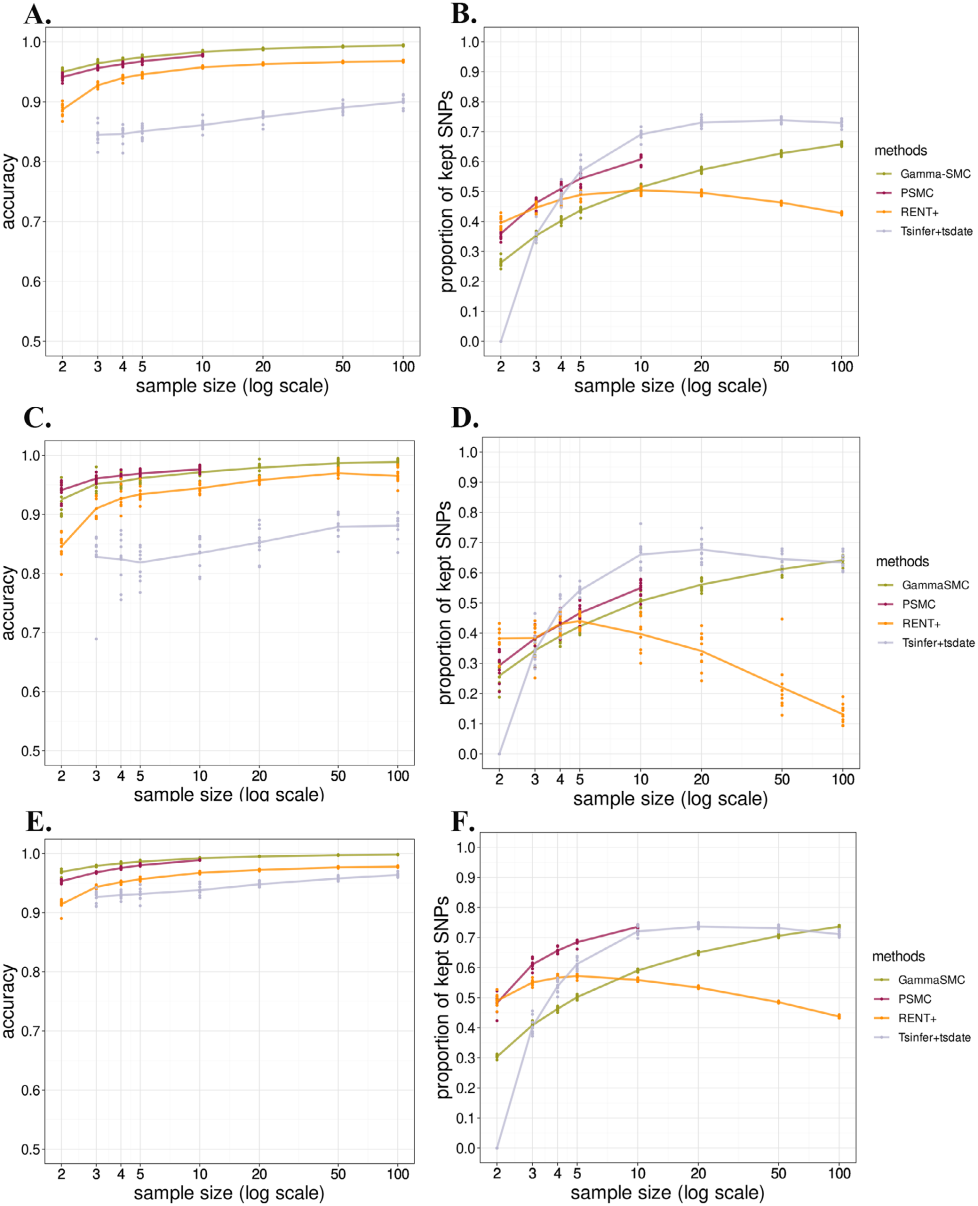
Polarising performance of PolarBEAR with inferred ARGs under different demographic scenarios. ARGs inference methods include gamma‐SMC, PSMC, RENT+, and tsinfer + tsdate, shown in different colours after applying filters. Comparison of total accuracy (A, C and E) and proportion of kept SNPs (B, D, and F) for different sample sizes. (A and B) Correspond to a constant population size; (C and D) To a decreasing population size; and (E and F) To an increasing population size. The points show 10 replicates in each group, and the lines represent their means. Missing values are reported when no sites are left after filtering.

We compared the accuracy of these methods after implementing all filters and found that the accuracy of gamma‐SMC, PSMC and RENT+ can reach more than 95% when the sample sizes are ten or more individuals, while tsinfer + tsdate only reaches 90% accuracy when the sample size is 100 (Figure 2A). The accuracy of all methods increases with the sample size, among which gamma‐SMC and PSMC have the highest accuracy (≥ 97% for a sample size of ten, with gamma‐SMC being slightly more accurate). When the true ancestral allele is the major one, all methods approach almost 100% accuracy (Figure S3A). When the frequencies of ancestral and derived alleles are equal, the variation in accuracy between different replicates of all methods becomes larger with increasing sample size because such sites become rare, especially after filtering is implemented. The advantage of gamma‐SMC is more pronounced when the true ancestral allele is the minor one, with an accuracy of 75% when the sample size is larger than ten. When the ancestral allele frequency is in the minority and half, the ranking of the accuracy of different methods is consistent with that of the total accuracy. On the other hand, PSMC has an advantage in the proportion of kept SNPs, at least in the case of small sample sizes (≤ 10), as it systematically retains 10% more sites than the gamma‐SMC method (Figure 2B). The poor performance of tsinfer + tsdate may be due to its reliance on the input ancestral allele. As we used the major allele as input ancestral state, it is incorrect for positions where the true ancestral allele is the minor one. This may explain why it has 100% accuracy when the ancestral alleles are the major ones but close to zero when the ancestral alleles are the minor ones (Figure S3A). We note that using the resulting ARG to re‐infer the ancestral alleles did not lead to any further improvement (Figure S4A,B). In contrast, when using the true ancestral alleles as input, the accuracy was above 90%, even when the ancestral allele was the minor one (Figure S4C). Moreover, when the sample size is two, unlike the case mentioned above where major alleles are used as input, inferred genealogies no longer display polytomies at the root nodes and the proportion of analysable sites increases from 0% to 36% (Figure S4D).

To test the performance of polarisation under different demographic scenarios, we simulated a logarithmic change of about 50 times within 150,000 generations for both the population size increase and decrease scenarios. When the population size decreases, the overall accuracy of the methods does not change significantly. However, PSMC, previously close to gamma‐SMC, now has the highest accuracy (Figure 2C). When the ancestral allele is the minor one, the accuracy of gamma‐SMC decreases more obviously, and that of PSMC exceeds it by more than 5% (Figure S3B). Furthermore, the proportion of SNPs kept after filtering of PSMC and RENT+ decreased with respect to the scenario of constant population size, while it did not change significantly for gamma‐SMC (Figure 2B,D). In the scenario where the population size increases, the overall accuracy of the methods is slightly higher than in the constant population size scenario (Figure 2A,E). However, this difference vanishes when classifying SNPs according to the frequency level of their ancestral alleles, suggesting that the average gain in accuracy is due to a higher proportion of SNPs where the ancestral allele is the major one in the population expansion scenario (Figure S3A,C). Furthermore, as opposed to when population size declines, the proportion of SNPs kept after filtering was higher for all methods in the growth scenario, with that of PSMC showing the largest increase (more than 10% increase, Figure 2B,F).

### 3.3 Impact of the Recombination Rate on the Ancestral Allele Reconstruction

The accuracy of ARG inference methods is known to be affected by the ratio of recombination rate to mutation rate, since recombination can only be detected when mutations are present on the segments that are separated by the recombination event. For a given mutation rate, the higher the recombination rate, the more recombination events will be unseen in the data. This dependency has been noted previously (Hayman et al. 2023). To understand how the recombination rate affects ARG inference and its impact on the polarisation results, we classified the SNPs according to the local recombination rate (see Methods). The accuracy ranking of the four methods was consistent with previous results, regardless of the recombination rate, and all methods had higher accuracy in regions with the lowest recombination rates, with tsinfer + tsdate having the smallest difference at 1% to 2% in accuracy between the two recombination rate classifications for all sample sizes (Figure S5A). The other three methods had greater differences in accuracy between low and high recombination rate regions, and these differences decreased with the sample size. For example, when the sample size was ten, the difference in accuracy between the two recombination rates of gamma‐SMC was more than 12%, that of PSMC was close to 14%, and it was up to 15% for RENT+; when the sample size was 100, this difference was only 1% for gamma‐SMC and RENT+. The sample size effect of the accuracy difference mainly comes from the improvement of accuracy at high recombination rates, while the accuracy under low recombination rates remains at a high level under most sample sizes: the accuracy of gamma‐SMC and PSMC is above 98% in all sample sizes, and that of RENT+ is above 95% when the sample size is greater than two.

The impact of the recombination rate is also stronger when the ancestral allele is the minor one: for a sample size of ten, the difference in accuracy between the two recombination rates of RENT+ reaches 27%, and for gamma‐SMC and PSMC it can even exceed 42% and 47%, respectively (Figure S5C). We speculate that this effect may be related to how topological inaccuracies translate into polarisation errors: the branches carrying a minor allele are more likely to be placed within a clade carrying the major allele, which does not impact the inference when the major allele is the ancestral one. Conversely, if the minor allele is the ancestral one, this leads to a mis‐polarisation. In other words, topological errors have a greater impact on ancestral allele reconstruction when the ancestor is the minor allele. As regions of high recombination rate have increased topological errors, they also exhibit higher mis‐polarisation when the ancestral allele is the minor one.

Recombination rate not only impacts the reconstruction accuracy but also the number of SNPs for which reconstruction can be performed. Some of the incorrectly reconstructed topologies will lead to a high number of inferred mutations and will be filtered out, leading to a lower proportion of kept SNPs. The proportion of analysable SNPs by gamma‐SMC and PSMC increases with the sample size for both recombination rate scenarios but increases less when the recombination rate is high (Figure S5B).

Among the tested ARG inference methods, local genealogies inferred using UPGMA on the matrix of pairwise TMRCAs inferred by gamma‐SMC offer the best compromise in accuracy, running speed, and memory usage. This approach notably permits the analysis of large sample sizes with good accuracy. When the sample size is not more than 10, PSMC is also a worthy alternative, given its accuracy close to that of gamma‐SMC and a 10% higher proportion of polarisable SNPs.

### 3.4 Estimating the Unfolded Site Frequency Spectrum

**FIGURE 3:**
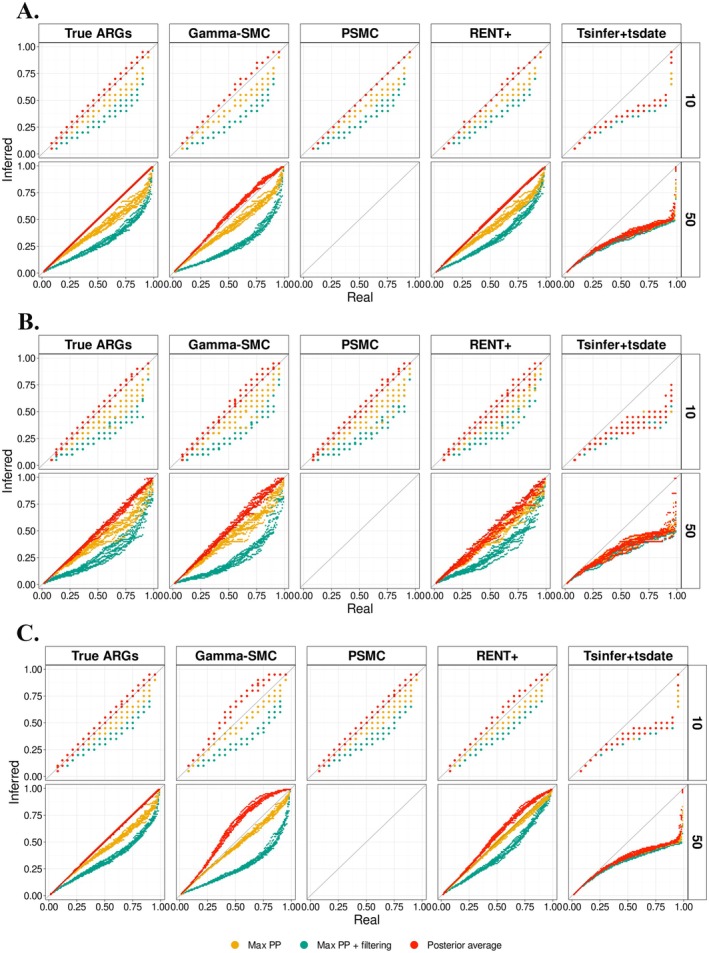
uSFS inference with different ARG reconstruction methods under different demographic scenarios. (A) Constant population size; (B) Decreasing population size; and (C) Increasing population size. The panels show QQ‐plots comparing inferred uSFS from different computation approaches (colours) with the true uSFS, using the cumulative proportions from the true uSFS as the reference to obtain corresponding quantiles. Columns correspond to different ARG reconstruction methods and rows correspond to sample sizes.

In this section, we present a scheme for estimating the unfolded site frequency spectrum (uSFS) using polarisation posterior probabilities. We compared three alternative methods to infer the uSFS and used quantile‐quantile (QQ) plots to compare the results with the true uSFS (Figures 3A and S6). Two methods directly use the polarisation results mentioned in the previous sections: with filtering (hereby referred to as the maximum posterior probability with filtering, “MAX PP + filtering” method); and without filtering of non‐informative genealogies (hereby referred to as the maximum posterior probability, “MAX PP” method). The uSFS inferred with the MAX PP + filtering method significantly deviates from the true uSFS, even more than the uSFS inferred without filtering. This is because the number of non‐informative genealogies depends on the derived allele frequencies, resulting in a bias when filtering genealogies (Figure S7).

We introduce a third method, hereby referred to as the “Posterior average” method. We include the uncertainty of ancestral allele reconstruction when computing the uSFS to account for possible polarisation errors. From the results in the previous sections, a source of uncertainty is non‐informative genealogies. We show that the relative branch lengths at the root node of these topologies contain information about the ancestral states, a signal captured by the posterior probabilities (Text S1). Using posterior probabilities as proxies for the polarisation error rate, we can compute the expectation of the true proportion of sites in each derived allele frequency (DAF) category. We consider a sample of n haplotypes, bi‐allelic polymorphic sites in DAF category $D_{x}$ with frequency xn have x copies of the derived allele and n−x copies of the ancestral allele. The expectation $E\left[D_{x}\right]$ of the true number of sites in category $D_{x}$ is then computed as the sum of probabilities of each SNP i to be in the category. For instance, a SNP with two A alleles and eight T alleles is in category $D_{2}$ if T is the ancestral allele and in category $D_{8}$ if A is the ancestral allele. The probability that the SNP belongs to category $D_{2}$, noted $P\left(D_{2}\right)$, is equal to the posterior probability that T is the ancestral state at this position. Similarly, $P\left(D_{8}\right)$, is equal to the posterior probability that A is the ancestral state at this position. Note that all other probabilities, $P\left(D_{1,3,4,5,6,7,9}\right)$ are null for this site.

We then have:

$$E\left[D_{x}\right]=\sum_{i}^{\;L_{x}}P_{i}\left(D_{x}\right),$$

where $L_{x}$ denotes the number of sites with allele frequencies $\left(x/n,\left(n-x\right)/n\right)$, in whichever order.

Using the posterior‐averaged uSFS with the true ARG leads to estimates that are only slightly biased in the high DAF categories, although with large variance for small sample sizes (Figures 3A and S6). This confirms that the branch length information in ARGs is included in the ancestral states posterior probabilities and can be used to provide an effective estimate for the uSFS.

We then examine the performance of four ARG inference methods (Figures 3A and S6). The genealogies with more than two mutations were filtered out because they were more likely to imply erroneous topologies and lead to incorrect posterior probabilities. We note $L_{x}^{\prime }$. the number of SNPs with allele frequencies $\left(x/n,\left(n-x\right)/n\right)$ that are kept after filtering. We then obtain the expectation of the true number of the SNPs in category $D_{x}$ with

$$E\left[D_{x}\right]=L_{x}\cdot \frac{1}{L_{x}^{\prime }}\sum_{i}^{\;L_{x}^{\prime }}P_{i}\left(D_{x}\right).$$

The uSFS count from ARGs inferred by gamma‐SMC, PSMC and RENT+ closely followed the QQ‐plot diagonal, but that of tsinfer + tsdate deviated significantly, possibly because of the incorrect input of major alleles as ancestral states. Among the first three methods, PSMC and RENT+ perform better, while the posterior average of gamma‐SMC is slightly overcorrected.

Since the shape of the uSFS is strongly impacted by demography, we assessed the uSFS estimation accuracy under the same simulations of population expansion and contraction scenarios as in section *Accuracy When the ARG Is Inferred from the Data*. Under the scenario of decreasing population size, we observed an increased variance in the uSFS estimations, possibly due to the reduction in the number of SNPs. However, the inferred uSFS was very close to the true one, the RENT+ method leading to the best inference in this scenario, in particular when the sample size was large (Figures 3B and S8). In the scenario of increasing population size, the uSFS obtained using the ARG inferred from PSMC was close to the true one, but the uSFS obtained from the ARGs inferred from RENT+ and gamma‐SMC was biased (Figures 3C and S9). We observe that (i) the performance ranking of ARG inference methods differs from the ranking when polarising SNPs, and (ii) for the inferred ARGs, although the MAX PP method appears to perform best under increasing population size, results based on the true ARGs indicate that MAX PP is in fact biased. We posit that this seemingly better performance results from a different bias that stems from gamma‐SMC and RENT+ ARG inference errors under this demographic scenario, the two opposite biases apparently compensating each other. Among the three methods, gamma‐SMC shows the largest departure from the true uSFS, possibly because it assumes a demographic model with constant population size in its flow field construction (Schweiger and Durbin 2023).

We note that, when using the true ARG, the uSFS is recovered with high accuracy by the posterior average method in all three demographic scenarios, suggesting that non‐constant population sizes affect the ARG reconstruction itself rather than the polarisation per se. Two properties of the inferred ARGs directly affect estimates of the uSFS: the proportion of inferred non‐informative genealogies and the accuracy of branch lengths. In informative genealogies, the polarisation accuracy is high (Figure 2A,C,E) and the maximum posterior probabilities are usually close to 1, so that the posterior average is very similar to the estimate based on the maximum posterior probability only. In non‐informative genealogies, however, the probabilities are closer to 0.5, reflecting the uncertainty in the ancestral state reconstruction and impacting the uSFS estimation. In these cases, the topologies of the clades at the root do not affect the inference, because the posterior probability depends only on the relative lengths of the two root branches under the chosen substitution model (see below and Text S1). While accounting for true non‐informative genealogies enables unbiased uSFS inference, incorrect reconstruction of non‐informative genealogies could lead to an over‐correction, especially when the number of SNPs in the two complementary categories $D_{x}$ and $D_{n-x}$ is very different. Furthermore, even when non‐informative genealogies are correctly recovered, underestimating the differences in branch lengths at the root node will push the posterior probability closer to 0.5, leading to a similar effect as overestimating the number of non‐informative genealogies. To understand the source of the bias in uSFS estimates caused by the results of different ARG inference methods, we examined the difference between the proportion of non‐informative genealogies from inference and the true proportion of non‐informative genealogies (Figure S7). Among the three demographic scenarios, the proportion of non‐informative genealogies inferred from PSMC is closest to the true one. The proportion of non‐informative genealogies estimated by gamma‐SMC is overestimated to different levels under the three demographic simulations: It is closest to the true one in the scenario of population contraction and greatly overestimated in the scenario of population expansion. In both cases, the overestimation is present in the most abundant low DAF categories. In contrast to gamma‐SMC, RENT+ underestimates the proportion of non‐informative genealogies under different demographic scenarios, an effect that increases with the sample size. However, in the scenario where the population size increases, the uSFS estimated using the results of RENT+ also shows a small amount of overcorrection compared to the MAX PP and MAX PP + filtering methods.

Since QQ‐plots focus on global distribution similarity, and the number of sites in the highest DAF categories is small, biases in these categories do not appear clearly with such representation. We compute the relative error, calculated as the difference between the inferred frequency and the true frequency, divided by the true frequency, to evaluate the performance of PolarBEAR with PSMC, the best‐performing method mentioned above (Figure S10). In the scenario of decreasing population size, when the sample size was ten, the number of sites in each DAF category was too small, so the variation was large, making it difficult to determine whether it was biased. When the sample size was five, however, the bias observed for the decreasing population size was the smallest among the three demographic scenarios. In the scenarios of constant and increasing population size, the high DAF categories were underestimated, which was most obvious in the last element, with relative errors of −0.16 and −0.13 for the two scenarios, respectively, when the sample size was ten. However, these biases were absent when the true ARGs were used, indicating that they are caused by the inaccuracy of ARG inference by PSMC.

In outgroup‐based uSFS estimation methods, misspecification of the substitution rate model will seriously affect the results (Keightley et al. 2016). It is worth mentioning that in our ARG‐based uSFS estimation, although we assume the Jukes‐Cantor model, the uSFS will not be biased when the ARG is accurate and the substitution rates between the two alleles are symmetric. In such a case, the posterior probability depends only on the ratio of the two branch lengths (Text S1). Conversely, the maximum posterior probabilities of the informative genealogies are close to one and the impact can be ignored. We verified this using the simulations with Kimura's two‐parameter model (Kimura 1980) with the transition/transversion rate ratio κ=5 (Figure S11). As expected, when the ARGs were known, the uSFS estimated by PolarBEAR was almost unbiased. When the ARGs were inferred from PSMC, the estimated uSFS was underestimated in the high DAF category, which is similar to the previous results and is probably due to inaccurate ARG reconstruction, but the relative error of uSFS did not differ among different mutation types.

The results in this section suggest that for the estimation of the uSFS based on the ARG, due to the uncertainty of the ancestral state at a specific site, directly using the most likely ancestral state will lead to a severe overestimation in low DAF categories and underestimation in high DAF categories, while combining with posterior averaging can well recover the uSFS. Additionally, the uSFS estimation using PSMC for ARG reconstruction performs best despite slightly underestimating the high DAF categories, followed by RENT+, while gamma‐SMC leads to a relatively large bias.

### 3.5 Effect of Background Selection on Polarisation and uSFS

To evaluate the impact of selection on ancestral reconstruction and, reciprocally, to assess the ability to infer selection from the uSFS, we used simulations based on Drosophila chromosome 2 L, where deleterious mutations were introduced in exonic regions alongside neutral sites using a realistic recombination map (see Methods). We then compared SNPs resulting from deleterious and neutral mutations.

**FIGURE 4:**
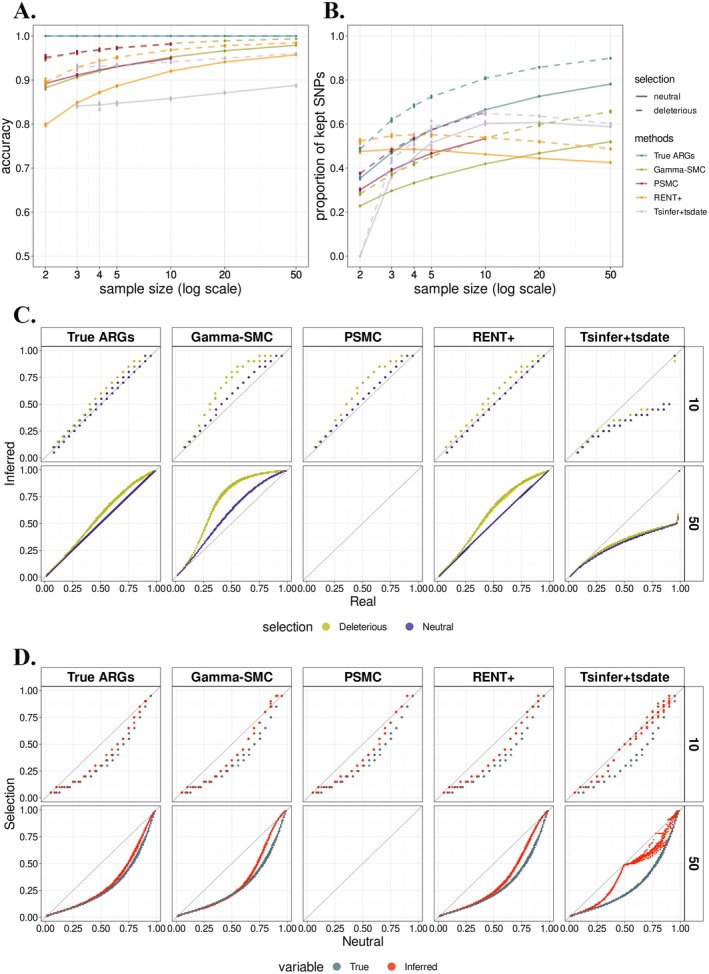
Impact of background selection on polarisation accuracy and uSFS estimation in PolarBEAR. (A and B) Comparison of total accuracy (A) and proportion of kept SNPs (B) for different sample sizes. Solid and dashed lines indicate neutral and deleterious mutations, respectively. Colours represent the true ARG and ARGs inferred by different methods. (C and D) The panels show the QQ‐plot comparing true and inferred uSFS (C) and comparing uSFS under neutral and purifying selection scenarios (D). Distinct colours indicate uSFS of sites carrying neutral versus deleterious mutations (C) and the true versus inferred uSFS (D). In each case, cumulative proportions are computed from one reference distribution (the true uSFS in C, and the uSFS of neutral sites in D), and the corresponding quantiles are then extracted from both distributions for comparison. Columns correspond to the true ARG and different ARG reconstruction methods, and rows correspond to sample sizes.

For SNP polarisation, when ARGs were inferred using four different methods, deleterious sites showed slightly higher accuracy than neutral ones after filtering (Figures 4A and S12). When the true ARG was used, however, the two classes of sites had identical and perfect accuracy, showing that the difference comes from the ARG inference rather than from the polarisation itself. We speculate that this is because lineages carrying deleterious alleles tend to leave fewer descendants, which increases the genetic distance between them and the rest of the sample, making local genealogies easier to resolve in ARG inference. At the same time, among SNPs with deleterious mutations, a larger fraction passed the filter compared to neutral SNPs, both when using the true ARG and inferred ones as input (Figure 4B), likely because under these simulation conditions, more sampled individuals carry the ancestral allele at selected sites, making it easier to form informative genealogies. In this setting, PSMC and gamma‐SMC achieved the highest and very similar accuracies (Figure 4A), with PSMC retaining more usable sites (Figure 4B).

For the uSFS, we first compared estimates by QQ plots as above. With the true ARG, neutral mutations aligned with the diagonal (Figures 4C and S13), showing no systematic bias and indicating that background selection on linked regions did not distort the neutral uSFS. Conversely, deleterious mutations showed a deviation. Since deleterious sites with informative genealogies polarised perfectly, we hypothesized that the bias was caused by non‐informative genealogies. To validate this, we separated deleterious SNPs according to whether their genealogies were informative and compared their relative errors against the true uSFS. For sites with informative genealogies, uSFS estimates were essentially unbiased across DAF categories, except for high‐frequency categories with few SNPs (Figure S14). For non‐informative genealogies, the proportion of SNPs in low DAF categories was underestimated, while it was overestimated in high DAF categories. We speculate that this is because deleterious alleles are often confined to lineages with fewer descendants towards the tip of the genealogy, violating the assumption that mutations occur randomly across branches and making the calculation of the likelihood and posterior probabilities incorrect.

Although the deleterious uSFS exhibited systematic deviations, signals of selection were not completely erased. Using QQ‐plots based on cumulative proportions of neutral uSFS, we found that the estimated spectrum of deleterious sites was closer to its true distribution than to the neutral expectation (i.e., the diagonal) (Figure 4D). This observation was made when the true ARG was used, but also held when using ARGs inferred by RENT+, PSMC, and gamma‐SMC. This may be explained by the above results that deleterious sites with informative genealogies were essentially unbiased, allowing the selection signal to be retained. Among the four ARG inference methods, the deleterious uSFS was recovered most accurately by RENT+, followed by PSMC, gamma‐SMC, and tsinfer + tsdate (Figure 4C). Overall, while PolarBEAR underestimates the impact of purifying selection in the presence of non‐informative genealogies, it still preserves detectable signals of selection, with performance depending on the proportion of informative genealogies and potentially influenced by the strength of selection.

### 3.6 Performance on Real Data

We compare PolarBEAR with the outgroup‐based method est‐sfs on a benchmark dataset. We used the human African population Mende, Sierra Leone (MSL) from the 1000 Genomes Project, containing 87 diploid individuals, restricting the analysis to chromosome 1. Orangutan and macaque were selected as outgroups for use with est‐sfs. Sites with missing data in the outgroups and non‐biallelic sites were discarded (Keightley and Jackson 2018), and the R6 model, allowing six symmetrical rates, was used to estimate the substitution rates. We used PolarBEAR with all SNPs that pass all quality criteria (see Methods). ARGs were inferred using gamma‐SMC and sites with non‐informative genealogies or an inferred number of mutations greater than the number of observed alleles were filtered out.

**FIGURE 5:**
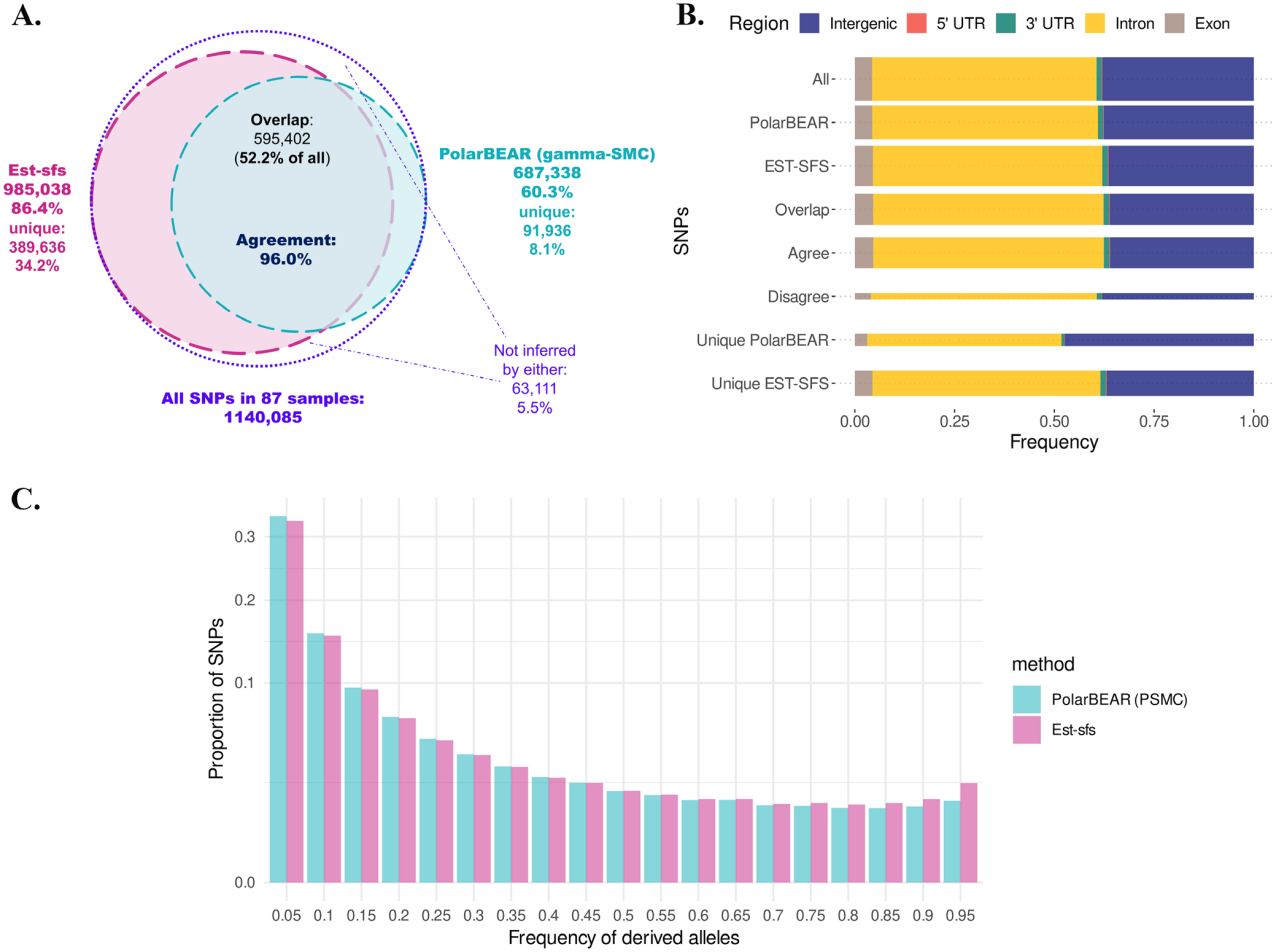
Comparison of est‐sfs and PolarBEAR polarisation of SNPs of human chromosome 1. (A) Numbers and proportions of SNPs that can be analysed by PolarBEAR, est‐sfs and their overlap, as well as the agreement in the overlap between the two methods. (B) Comparison of SNPs frequencies and methods agreement, in exon, intron, 5′ UTR, 3′UTR and intergenic regions. The widths of the boxes are proportional to the square roots of the numbers of SNPs in the groups. (C) USFS estimated by est‐sfs (pink) and PolarBEAR with PSMC (blue). The *y*‐axis (SNP proportion) is scaled using a square root transformation.

We first examined the proportion of SNPs that were polarisable by each method. Then, among the SNPs polarisable by both methods, we focused on the proportion where the polarisation results agreed to evaluate the consistency between the two methods. est‐sfs could analyse 86.4% of the input SNPs, while PolarBEAR could analyse a proportion of 60.3% of them. 52.2% of the SNPs could be analysed by both methods, and 5.5% could not be analysed by either PolarBEAR or est‐sfs (Figure 5A). The two methods returned the same ancestral allele in 96.0% of the SNPs that could be analysed by both methods. Using PolarBEAR in conjunction with est‐sfs brings the proportion of polarisable SNPs from 86.4% to 94.5%.

To examine the putative effect of selection on the performance of the two methods, we compared the SNPs in different regions expected to have different levels of selective pressure (Figure 5B). SNPs were classified into exons, 5′ UTRs, 3′ UTRs, introns, and intergenic regions. If selection affects the inference of PolarBEAR more than the reference method est‐sfs, we expect that the distribution of SNPs across genomic regions will differ when comparing the set of SNPs for which the two methods provide consistent results and the complete set of SNPs analysable by both methods. In particular, such consistently polarised SNPs should be under‐represented in exonic regions, where selection is the strongest. First, while significantly different, the distribution of SNPs polarisable by PolarBEAR and est‐sfs is very close to the global genome distribution (Figure 5B, chi2 test, *p* value = 6.647e‐05 for PolarBEAR and 2.2e‐16 for est‐sfs). We note that the SNP distribution of est‐sfs differs from the genome distribution to a larger extent than that of PolarBEAR. This difference is mostly attributable to a lower proportion of analysable SNPs in intergenic regions. However, the frequency of agreement does not differ between genomic regions (chi square test, distribution of SNPs where the two methods give a consistent result compared to the distribution of SNPs analysable by both methods, *p* value = 0.8742). A comparatively higher fraction of positions cannot be aligned with the outgroup or may display alignment errors in intergenic regions, as they evolve faster than coding regions. Based on these results, selection does not appear to be a major confounding factor for polarisation using the ARG in this dataset.

We inferred the uSFS with PolarBEAR using the ARG obtained with PSMC, as it was shown to provide the best performance. Sites that were inferred to have more than two mutations were excluded. We randomly selected ten individuals from the MSL population. In the middle part of the uSFS (DAF 0.3–0.7), PolarBEAR and est‐sfs showed a strong agreement, but PolarBEAR inferred higher frequencies for low‐frequency derived allele categories, whereas est‐sfs inferred an increase in the frequencies of high‐frequency derived variants (Figure 5C). Similar to the relative error calculated above, the uSFS from est‐sfs was used as the “standard” to show the relative difference between PolarBEAR and est‐sfs (Figure S16, black). Values of relative difference decreased with DAF and were negative in high DAF categories. Although part of it can be explained by the underestimation of PolarBEAR with PSMC, in the simulations with three different demographic scenarios and the same sample size of ten, none of the last elements in the DAF among all replicates has a relative error greater than −0.26 (Figure S10), contrasting with the relative difference of −0.32 observed in real data. This points to a potential additional source of discrepancy between the two methods. To examine whether the differences could be due to selection, we divided the SNPs into annotation categories such as exons, 5′ UTRs, 3′ UTRs, introns, and intergenic regions as mentioned in the polarisation above and estimated uSFS for different regions. Since est‐sfs only outputs uSFS based on all sites, we re‐estimated the uSFS in different categories using the site‐specific posterior probabilities from its output (see Methods). However, similar to the results of the polarisation section, the differences between the two methods did not show a clear correlation with the expected level of selection intensity in these regions (Figure S16), implying that selection was not the source of the differences in the uSFS from the two methods.

To examine the effect of mutation type on uSFS inference, we oriented mutation types assuming the ancestral state inferred by est‐sfs to be true and then compared the relative differences in uSFS estimated by the two methods across mutation types (Figure S17). The relative differences of uSFS of different mutation types showed the same trend as that of the global, that is, the proportion of variants estimated by PolarBEAR in the high DAF category was smaller than that estimated by est‐sfs. What's more, “A‐ > G” and “T‐ > C” together showed a more obvious difference between the two methods; while the other types had much smaller differences, suggesting that the reason for the difference between the two methods is that at least one of the estimations is sensitive to the rates of directional substitutions. When looking at the two methods separately, we found that the estimated uSFS for different mutation types differed only in est‐sfs, and the difference was that in “A‐ > G” and “T‐ > C”, a higher proportion of variants were estimated in high DAF categories, but not with PolarBEAR (Figure S18). Unexpectedly, in simulation studies with the presence of only an unequal GC‐to‐AT content ratio of 3:2 in the ancestral distribution, without involving differences in substitution rates, we found that it can also differentially bias est‐sfs estimates for different mutation types (Figure S19). As done for real data, when the polarisation results from est‐sfs were used to determine the mutation type of the SNPs, the relative differences between the two methods became similar to the differences observed in the real data across mutation types (Figure S20). We note, however, that despite the bias observed for certain mutation types in the simulation, the global uSFS was recovered correctly. Further parameter combinations must be assessed to further characterise the est‐sfs bias in the real data.

## 4 Conclusions

We show that the ARG intrinsically contains information about the ancestral allele, which can be unravelled using ARG inference methods and a probabilistic ancestral sequence reconstruction framework. This framework, which we named PolarBEAR, achieved similar performance to outgroup‐based methods where comparable, and greater performance than simply considering the major allele as ancestral in all scenarios that we tested.

Dissecting the performance of PolarBEAR, we showed that it mostly depends on the underlying genealogy topologies, which we classified as informative and non‐informative. Positions with non‐informative genealogies do not permit inference with high confidence, but we show that posterior probabilities effectively leverage the branch length information to capture the probabilities of ancestral states and that a posterior averaging procedure can be employed to infer the unfolded site frequency spectrum with good accuracy. While a bias persists for mutations under selection, the estimation is unbiased for neutral mutations, even in the presence of linked selection. Therefore, PolarBEAR offers a powerful approach to infer the neutral SFS: regions putatively under selection, such as coding region, can be discarded while linked regions can be safely kept. Conversely, extra caution should be given when the reconstructed SFS is to be used for inferring the patterns of selection.

Our result demonstrates the power of ARG‐based polarisation and uSFS estimation, but also that inference is impacted by errors in the ARG reconstruction. We found that using SMC methods to infer pairwise TMRCA values, and then employing a UPGMA tree reconstruction, provides good results under the set of conditions tested. For large sample sizes, heuristics like RENT+ can be employed. We posit that the accuracy of outgroup‐free polarisation will further increase with the improvement of ARG inference methods, including newer approaches such as SINGER (Deng et al. 2024). Another potential (yet more computationally demanding) avenue for improvement is to extend PolarBEAR to incorporate posterior distributions of ARGs, rather than conditioning on a fixed ARG.

While PolarBEAR could polarise fewer sites than est‐sfs on our benchmark dataset, it could analyse SNPs where no outgroup sequence was available. Furthermore, the non‐usage of outgroups reduces the issue of substitution model misspecification, which biases uSFS estimates. Therefore, PolarBEAR can serve as a powerful alternative or complementary method for polarising SNPs.

## Author Contributions

J.L.: conceptualization, methodology, software, formal analysis, investigation, data curation, writing – original draft, writing – review and editing, visualisation. J.Y.D.: conceptualization, methodology, writing – review and editing, supervision.

## Conflicts of Interest

The authors declare no conflicts of interest.

## Supporting information


**Supp. Pseudocode 1:** Inferring ancestral states for a single local tree.
**Supp. Text 1:** Branch lengths influence the posterior probabilities of ancestral alleles in non‐informative genealogies.
**Figure S1:** Polarising performance of PolarBEAR for sites with multiple mutation events with simulated ARG.
**Figure S2:** Effects of different filtering schemes on the performance of PolarBEAR with ARGs inferred by different methods.
**Figure S3:** Polarising performance of PolarBEAR with inferred ARGs for three demographic scenarios.
**Figure S4:** Polarising performance of PolarBEAR with tsinfer + tsdate using the first round of polarisation results with all filters (A and B) and true ancestral alleles (C and D) as input.
**Figure S5:** Effects of high and low recombination rates (shown in dashed and solid lines) on the performance of PolarBEAR with ARGs inferred by gamma‐SMC, PSMC, RENT+, and tsinfer + tsdate (shown in different colors).
**Figure S6:** uSFS inference with different ARG reconstruction methods under a constant demography scenario.
**Figure S7:** Proportion of SNPs with non‐informative genealogies as a function of the derived allele frequency.
**Figure S8:** uSFS inference with different ARG reconstruction methods under a decreasing population size scenario.
**Figure S9:** uSFS inference with different ARG reconstruction methods under an increasing population size scenario.
**Figure S10:** Relative error of uSFS inference from simulations.
**Figure S11:** Relative error of uSFS estimated from simulations under distinct transition and transversion rates.
**Figure S12:** Effects of purifying selection on the polarisation accuracy of PolarBEAR with ARGs inferred by gamma‐SMC, PSMC, RENT+, and tsinfer + tsdate (shown in different colors).
**Figure S13:** uSFS inference for sites with neutral and deleterious mutations (colours).
**Figure S14:** Relative error of uSFS estimated from sites under purifying selection with non‐informative or informative genealogies.
**Figure S15:** QQ‐plots comparing uSFS under neutral and purifying selection scenarios.
**Figure S16:** Relative difference between uSFS estimated by PolarBEAR and est‐sfs in different genomic regions.
**Figure S17:** Relative difference between uSFS estimated by PolarBEAR and est‐sfs, for distinct mutation types.
**Figure S18:** Estimated uSFS for different mutation types.
**Figure S19:** Relative error of uSFS estimated from simulations under unequal GC content.
**Figure S20:** Relative differences between uSFS estimated by PolarBEAR and est‐sfs under unequal GC content.

## Data Availability

All scripts used in this work are available at https://gitlab.gwdg.de/molsysevol/polarizing_without_outgroup. These scripts can be used with other datasets to polarise SNPs and infer uSFS from VCF files.
