# Supplementary material for: Polarising SNPs Without Outgroup
Source: Mol Ecol Resour. 2026 Feb 6;26(2):e70105. doi: 10.1111/1755-0998.70105 (PMC12878804; doi:10.1111/1755-0998.70105)
Supplement: Supplementary file 1 — Supp. Pseudocode 1: Inferring ancestral states for a single local tree. Supp. Text 1: Branch lengths influence the posterior probabilities of ancestral alleles in non‐informative genealogies. Figure S1: Polarising performance of PolarBEAR for sites with multiple mutation events with simulated ARG. Figure S2: Effects of different filtering schemes on the performance of PolarBEAR with ARGs inferred by different methods. Figure S3: Polarising performance of PolarBEAR with inferred ARGs for three demographic scenarios. Figure S4: Polarising performance of PolarBEAR with tsinfer + tsdate using the first round of polarisation results with all filters (A and B) and true ancestral alleles (C and D) as input. Figure S5: Effects of high and low recombination rates (shown in dashed and solid lines) on the performance of PolarBEAR with ARGs inferred by gamma‐SMC, PSMC, RENT+, and tsinfer + tsdate (shown in different colors). Figure S6: uSFS inference with different ARG reconstruction methods under a constant demography scenario. Figure S7: Proportion of SNPs with non‐informative genealogies as a function of the derived allele frequency. Figure S8: uSFS inference with different ARG reconstruction methods under a decreasing population size scenario. Figure S9: uSFS inference with different ARG reconstruction methods under an increasing population size scenario. Figure S10: Relative error of uSFS inference from simulations. Figure S11: Relative error of uSFS estimated from simulations under distinct transition and transversion rates. Figure S12: Effects of purifying selection on the polarisation accuracy of PolarBEAR with ARGs inferred by gamma‐SMC, PSMC, RENT+, and tsinfer + tsdate (shown in different colors). Figure S13: uSFS inference for sites with neutral and deleterious mutations (colours). Figure S14: Relative error of uSFS estimated from sites under purifying selection with non‐informative or informative genealogies. Figure S15: QQ‐plots comparing uSFS under neutral and pu [file MEN-26-e70105-s001.pdf]

Supplementary Information

Polarising SNPs without outgroup

Jinyang Liang[1, *], Julien Y. Dutheil[1]

[1]Department of Theoretical Biology, Max Planck Institute for Evolutionary Biology,

August-Thienemann-Straße 2, 24306 Plön, GERMANY

*Corresponding author: Email: jliang2@ed.ac.uk

```
FUNCTION calculate_likelihood(tree, node, genotypes, theta):
    # Step 1: If the node is a leaf, return conditional likelihood
    IF tree.is_leaf(node):
        likelihood = [0, 0, 0, 0]   # Initialize likelihood for A, C, G, T
        likelihood[genotypes[node]] = 1   # Set observed genotype likelihood to 1
        RETURN likelihood

    # Step 2: If not a leaf, process children
    children = tree.children(node)

    # Step 3: Traverse children and compute likelihood recursively
    FOR each child IN children:
        # Recursively calculate child likelihood
        child_likelihood = calculate_likelihood(tree, child, genotypes, theta)
        branch_length = tree.branch_length(child)

        # Step 4: Compute likelihood for each nucleotide type at the current node
        FOR state FROM 0 TO 3:   # Iterate over nucleotide types (A, C, G, T)
            max_prob = -INF
            FOR prev_state FROM 0 TO 3:   # Previous nucleotide state in the child
                # No mutation
                IF state == prev_state:
                    transition_prob = exp(-branch_length * theta)
                # Mutation occurs
                ELSE:
                    transition_prob = (1/3) * (1 - exp(-branch_length * theta))
                # Use log probabilities to prevent underflow
                IF 0 IN child_likelihood:
                    max_prob = log(MAX(transition_prob * child_likelihood[prev_state]))
                ELSE:
                    prob = log(transition_prob) + child_likelihood[prev_state]
                    max_prob = MAX(max_prob, prob)

            # Step 5: Update likelihood for the current nucleotide type
            likelihood[state] += max_prob

    # Step 6: Return the computed likelihood matrix for this node
    RETURN likelihood

FUNCTION normalize_likelihood_matrix (log_likelihood_matrix):
    max_log_likelihood = MAX(log_likelihood)
    likelihoods = [exp(lik - max_log_likelihood) FOR lik IN log_likelihood]
    sum_likelihoods = SUM(likelihoods)
    normalized_likelihood = [lik / sum_likelihoods FOR lik IN likelihoods]
    RETURN likelihood

FOR genealogy IN ARGs:
    log_likelihood_matrix = calculate_likelihood(genealogy, tree_root, genotypes, theta)
    posterior_probabilities = normalize_likelihood_matrix(log_likelihood_matrix)

    max_prob = MAX(posterior_probabilities)
    inferred_state = ARGMAX(posterior_probabilities)
```

Supp. Pseudocode 1: Inferring ancestral states for a single local tree. Recursively find the leaf nodes from the local genealogies at segregating sites, initialize the likelihood matrix with the genotypes in the samples, use the mutation rate and branch lengths as factors for the probabilities that mutations occur or do not occur, and calculate upward layer by layer. Finally, return to the root node and obtain the genotype with the greatest posterior probability.

Supp. Text 1: Branch lengths influence the posterior probabilities of ancestral alleles in non-informative genealogies

If we assume a non-informative genealogy:

1. two branches are starting from the root node connecting to the nodes N_1 and N_2 with branch lengths of L_1 and L_2;

2. the genotype of all samples under N_1 is G_1, and the genotype of all samples under N_2 is G_2;

3. the rate of changing from G_1 to G_2 is equal to the rate of changing from G_2 to G_1, denoted as θ.

The probability of a mutation of a certain type is denoted as

$$\mathrm{Mut}(BranchLength, \theta) = \frac{1}{3} \cdot \left(1 - e^{-BranchLength \cdot \theta}\right)$$

The probability of mutation doesn’t occur is denoted as

$$\mathrm{NoMut}(BranchLength, \theta) = e^{-BranchLength \cdot \theta}$$

Because the mutation rate is low, the likelihood that the genotypes at two nodes are the observed genotypes in their descendant samples is close to 1. Under the infinite site model, the mutation must have occurred in one of the two branches from the root, so there couldn't be any mutation under the N_1 and N_2 nodes. So, the observed genotype G_1 and G_2 at N_1 and N_2 can be seen as given conditions. Instead of Polarising along the entire tree, it can be viewed as Polarising only along a tree with a root node and two "ancient samples" (N_1 and N_2) with different branch lengths.

So, the probability of G_1 as genotype of the root is:

$$\mathrm{P1} \approx \mathrm{NoMut}(L_1, \theta) \cdot \mathrm{Mut}(L_2, \theta)$$

the probability of G_2 as genotype of the root is:

$$\mathrm{P2} \approx \mathrm{Mut}(L_1, \theta) \cdot \mathrm{NoMut}(L_2, \theta)$$

Expand $\mathrm{Mut}(BranchLength, \theta)$ and $\mathrm{NoMut}(BranchLength, \theta)$:

$$\mathrm{P1} = \mathrm{NoMut}(L_1, \theta) \cdot \mathrm{Mut}(L_2, \theta)$$

$$= e^{-L_1 \cdot \theta} \cdot 1/3 \cdot (1 - e^{-L_2 \cdot \theta})$$

$$= 1/3 \cdot (e^{-L_1 \cdot \theta} - e^{-(L_1+L_2) \cdot \theta})$$

similarly,

$$\mathrm{P2} = \mathrm{Mut}(L_1, \theta) \cdot \mathrm{NoMut}(L_2, \theta) = 1/3 \cdot (e^{-L_2 \cdot \theta} - e^{-(L_1+L_2) \cdot \theta})$$

So, the ratio of P1 to P2 is,

$$\frac{\text{P1}}{P2} = \frac{e^{-L_1 \cdot \theta} - e^{-(L_1+L_2)\cdot\theta}}{e^{-L_2\cdot\theta} - e^{-(L_1+L_2)\cdot\theta}}$$

Divided both P1 and P2 by $e^{-(L_1+L_2)\cdot\theta}$,

$$\frac{\text{P1}}{P2} = \frac{e^{(-L_1+L_1+L_2)\cdot\theta} - 1}{e^{(-L_2+L_1+L_2)\cdot\theta} - 1} = \frac{e^{L_2\cdot\theta} - 1}{e^{L_1\cdot\theta} - 1}$$

When $L_2 \cdot \theta \to 0$ *and* $L_1 \cdot \theta \to 0$:

$$e^{L_2\cdot\theta} \cong L_2 \cdot \theta + 1$$

$$e^{L_1\cdot\theta} \cong L_1 \cdot \theta + 1$$

so,

$$\frac{\text{P1}}{P2} \cong \frac{L_2 \cdot \theta}{L_1 \cdot \theta} = \frac{L_2}{L_1}$$

If $L_2 > L_1$, G_1 is the ancestral state, with posterior probability $P \cong L_1/(L_1 + L_2)$.

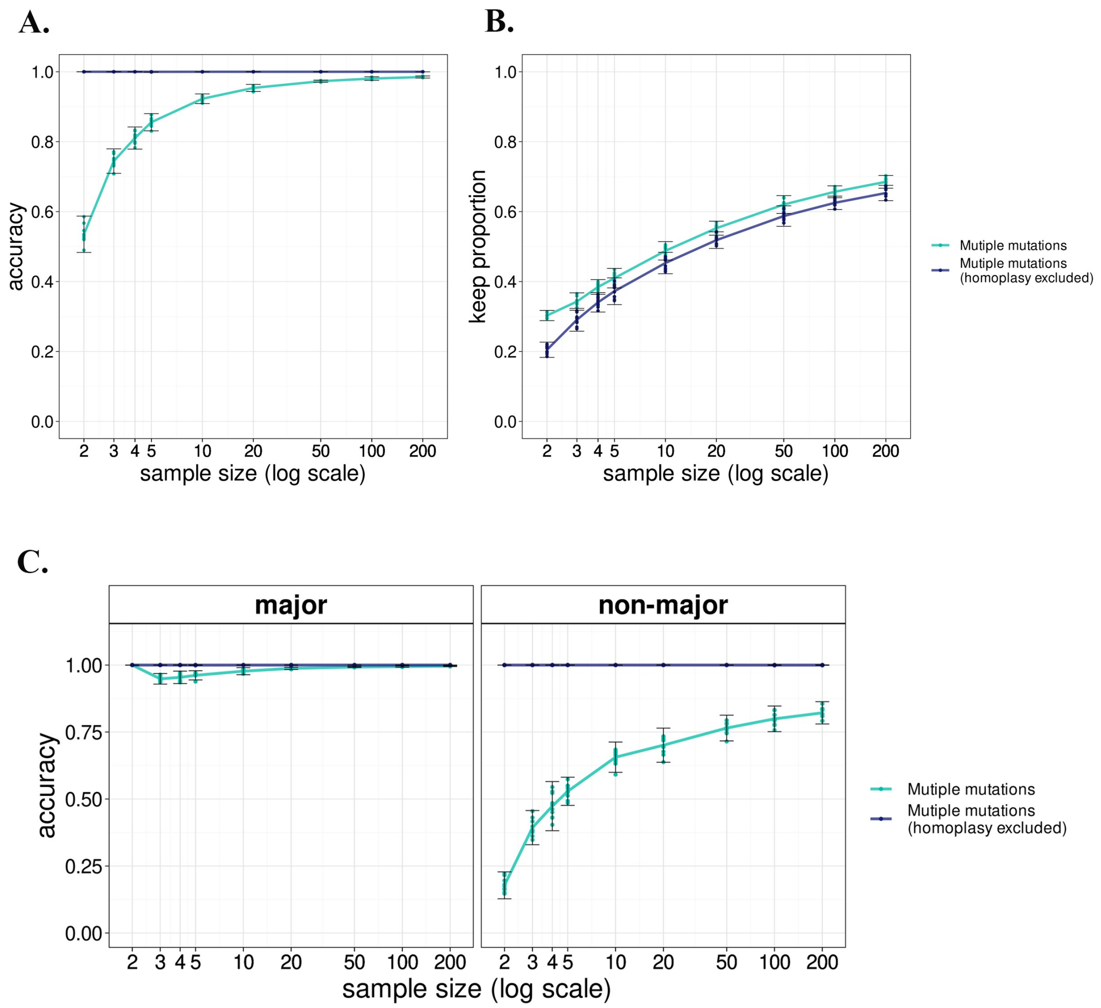

Figure S1. Polarising performance of PolarBEAR for sites with multiple mutation events with simulated ARG. Total accuracy (A), proportion of kept SNPs (B) and accuracy among the SNPs classified by their ancestral alleles as major and minor (C) for different sample sizes, with and without excluding the homoplasy sites. The points show 10 replicates in each group, and the lines represent their means.

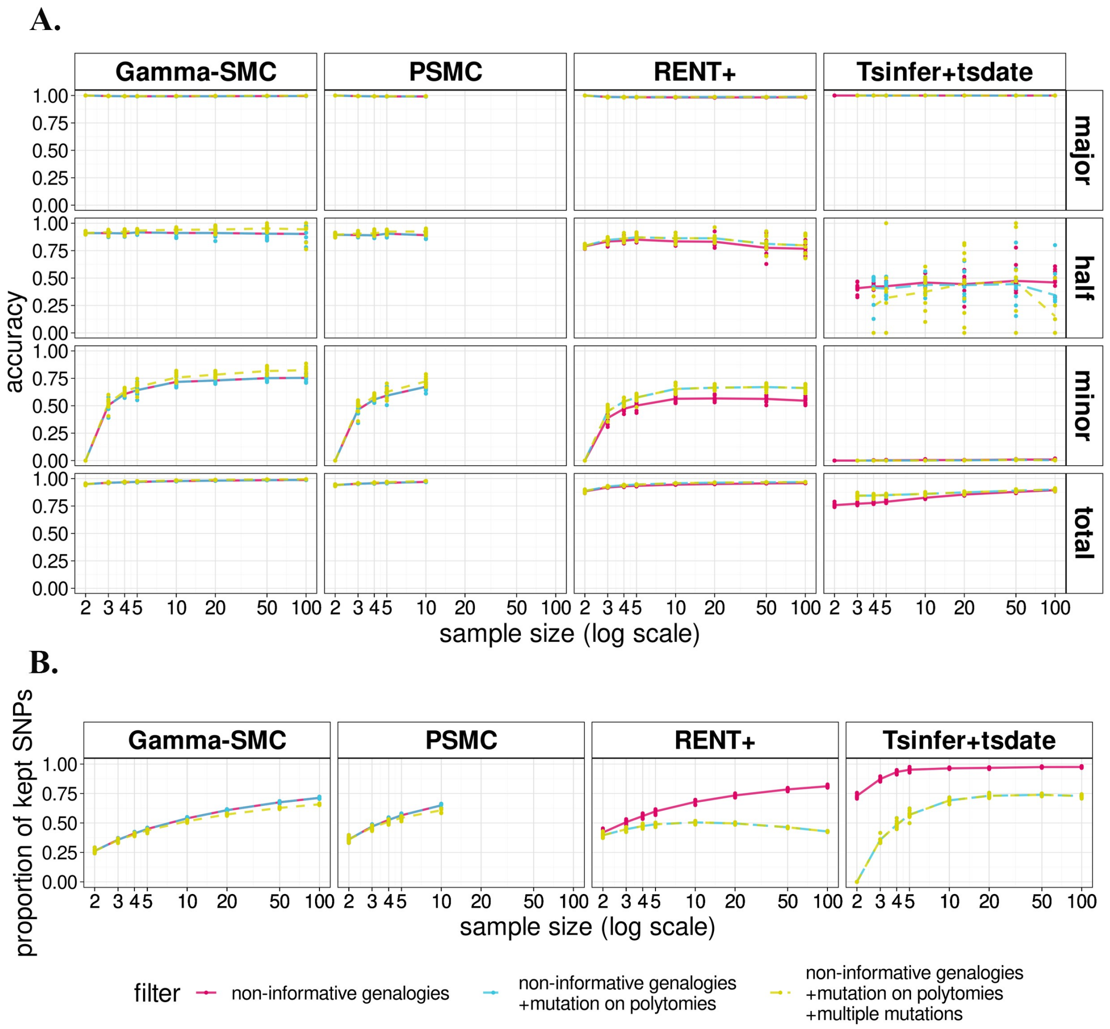

Figure S2. Effects of different filtering schemes on the performance of PolarBEAR with ARGs inferred by different methods. Filter 1: only non-informative genealogies are filtered. Filter 2: Filter 1 + sites where mutations occur at polytomies are filtered. Filter 3: Filter 2 + sites that require more than one mutation (as inferred by maximum parsimony reconstruction) to explain the distribution of the genotypes on leaves are filtered, represented by different colours. Accuracy comparison for SNPs classified by their ancestral allele frequencies (major, equal, or minor, A), and proportion of kept SNPs (B) for different sample sizes. The points show 10 replicates in each group, and the lines represent their means.

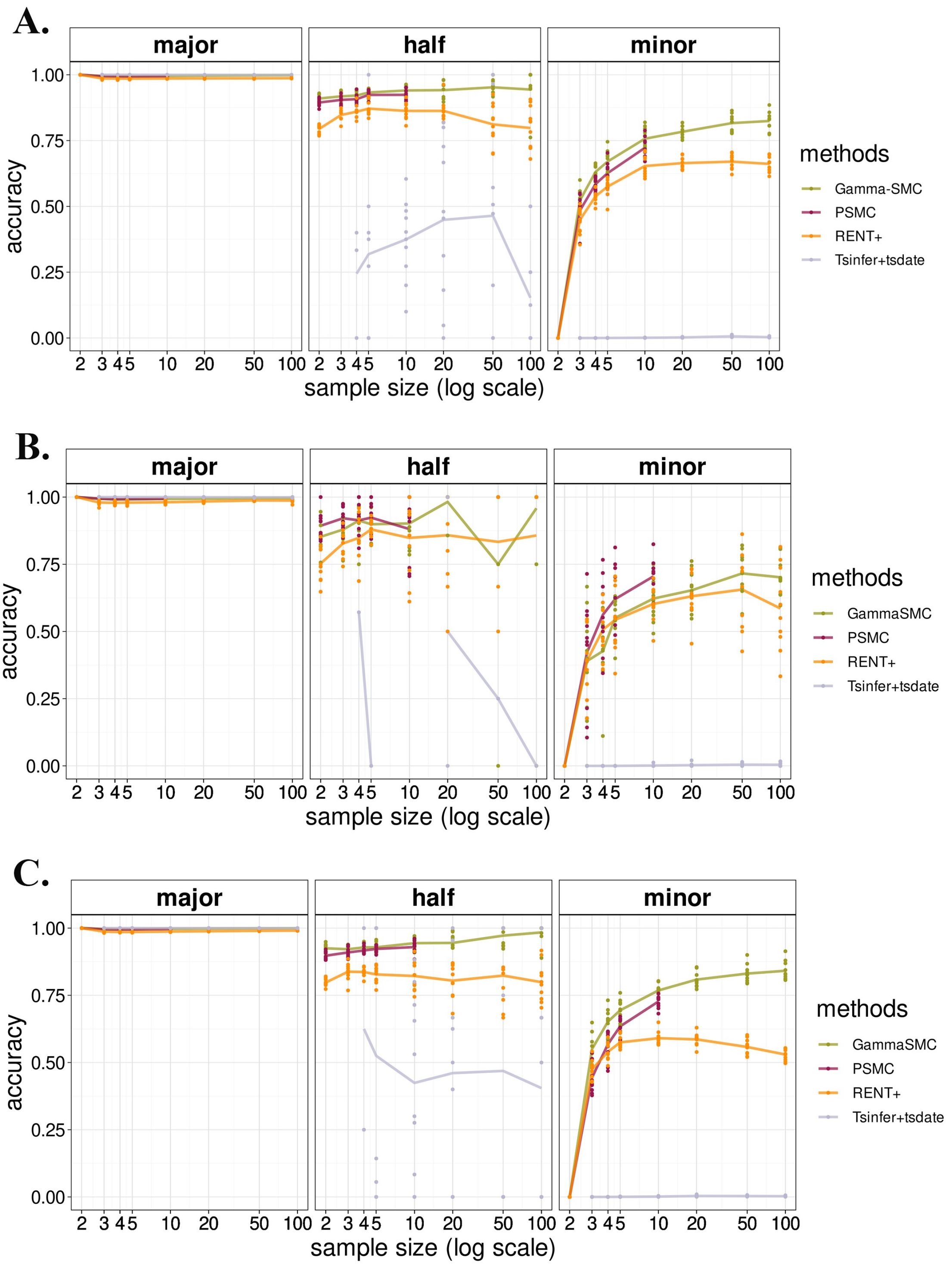

Figure S3. Polarising performance of PolarBEAR with inferred ARGs for three demographic scenarios. Accuracy of SNPs classified according to their ancestral alleles' frequencies (major, equal, and minor, for a constant (A), a declining (B) and an increasing (C) population size scenario, for different sample sizes. The points show 10 replicates in each group, and the lines represent their means.

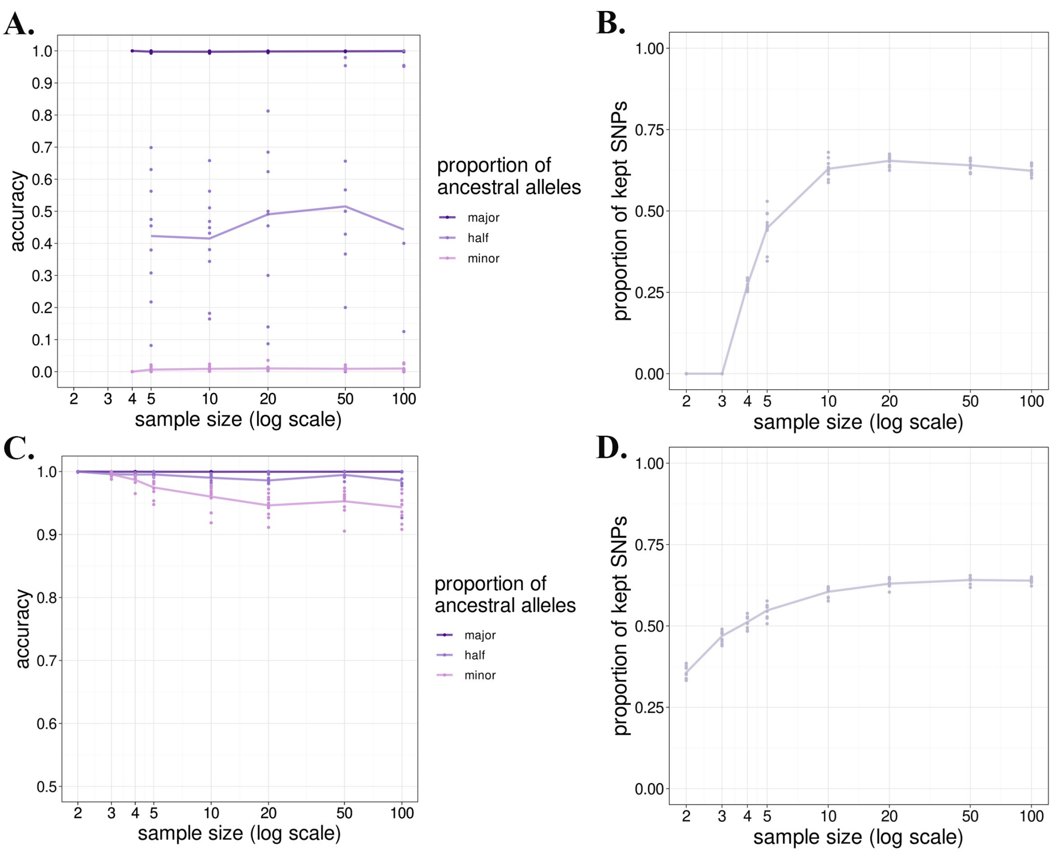

Figure S4. Polarising performance of PolarBEAR with tsinfer+tsdate using the first round of Polarisation results with all filters (A and B) and true ancestral alleles (C and D) as input. Comparison of Polarisation accuracy of SNPs classified by the frequency of their ancestral alleles (major, equal, or minor) shown in different colours (A and C) and proportion of analyzed SNPs after filtering (B and D) for different sample sizes.

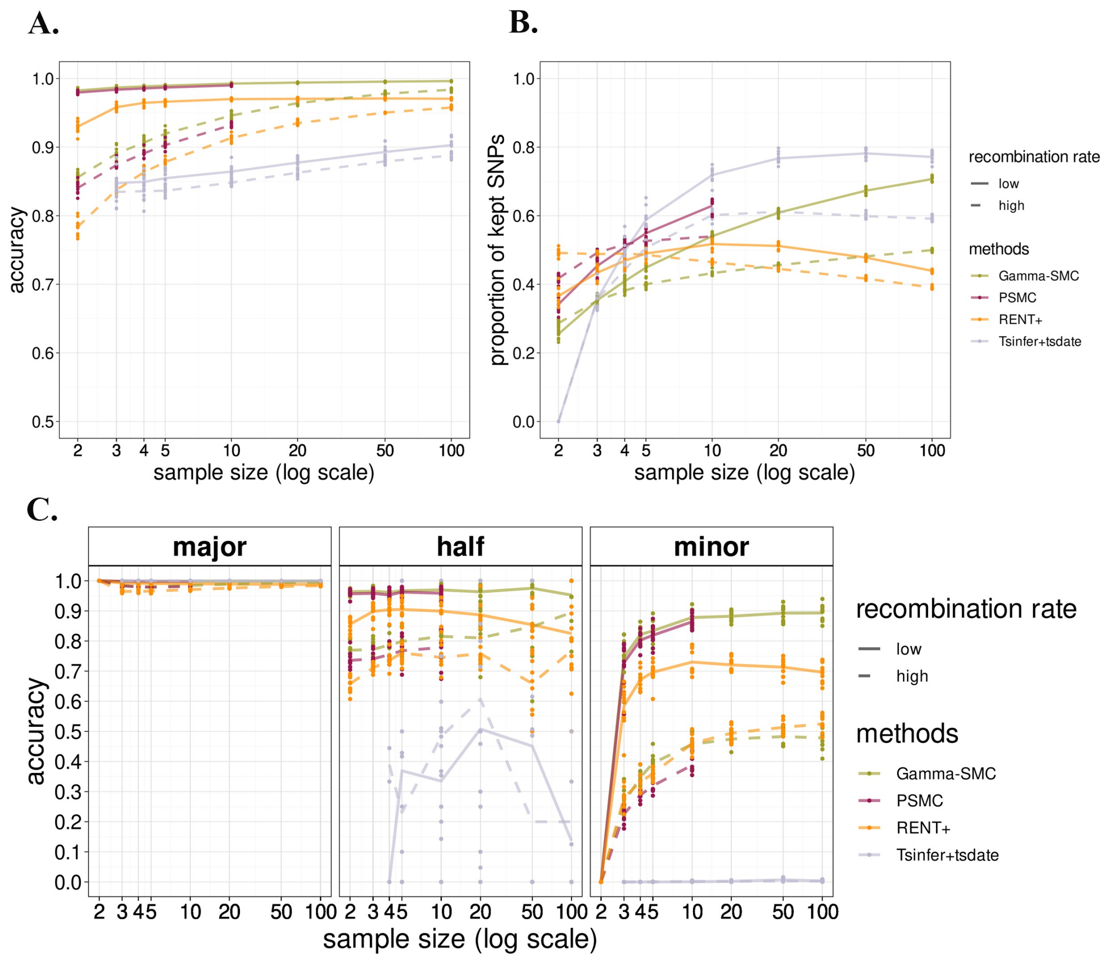

Figure S5. Effects of high and low recombination rates (shown in dashed and solid lines) on the performance of PolarBEAR with ARGs inferred by gamma-SMC, PSMC, RENT+, and tsinfer+tsdate (shown in different colours). Comparison of total accuracy (A), proportion of analyzed SNPs after filtering (B) and accuracy for SNPs classified by their ancestral allele frequency (major, equal, or minor, C) for different sample sizes. The points show 10 replicates in each group, and the lines represent their means.

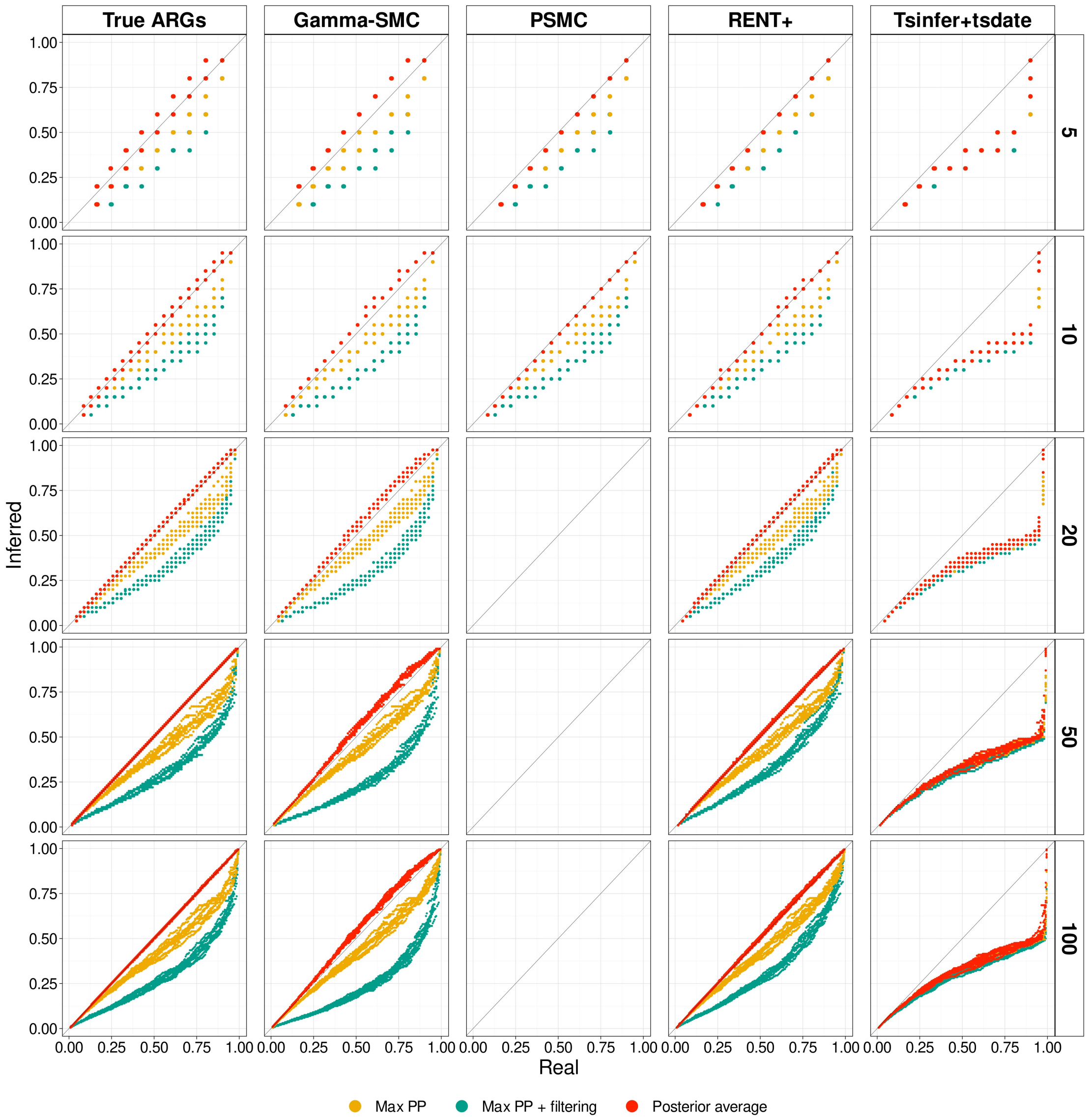

Figure S6. uSFS inference with different ARG reconstruction methods under a constant demography scenario. The panels show QQ-plots comparing inferred uSFS from different computation approaches (colours) with the true uSFS, using the cumulative proportions from the true uSFS as the reference to obtain corresponding quantiles. Columns correspond to different ARG reconstruction methods and rows correspond to sample sizes.

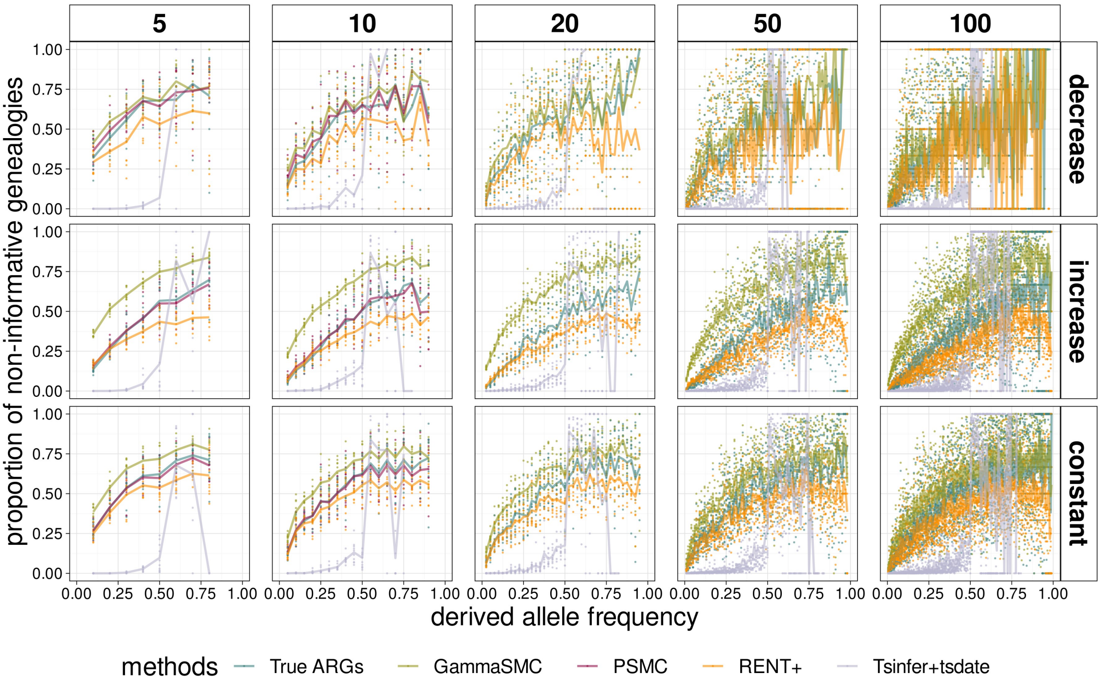

Figure S7. Proportion of SNPs with non-informative genealogies as a function of the derived allele frequency. Ancestral alleles were inferred by PolarBEAR with gamma-SMC, PSMC, RENT+, and tsinfer+tsdate (in different colours), for different sample sizes (in different columns) and under different demographic scenarios (in different rows).

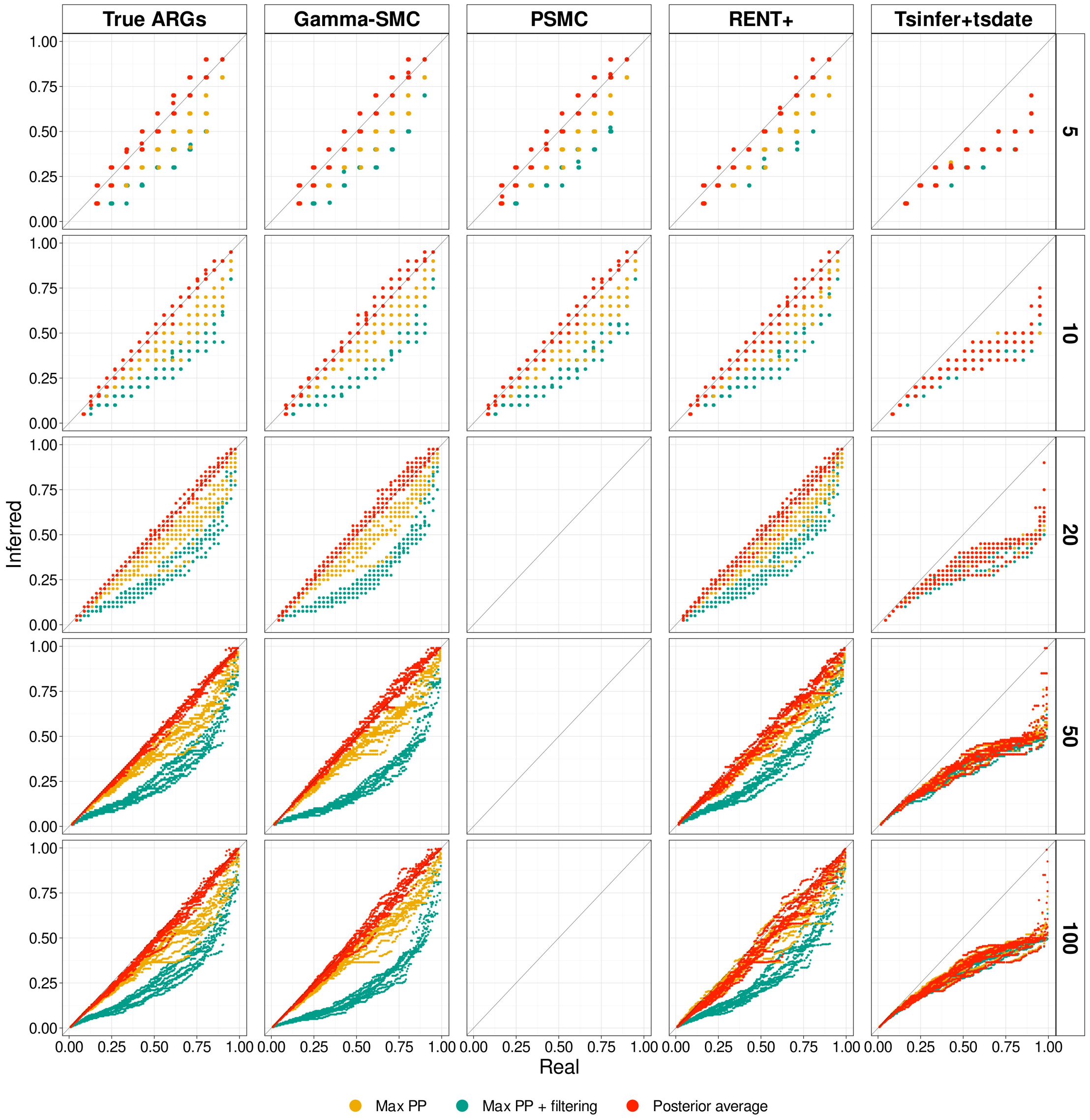

Figure S8. uSFS inference with different ARG reconstruction methods under a decreasing population size scenario. The panels show QQ-plots comparing inferred uSFS from different computation approaches (colours) with the true uSFS, using the cumulative proportions from the true uSFS as the reference to obtain corresponding quantiles. Columns correspond to different ARG reconstruction methods and rows correspond to sample sizes.

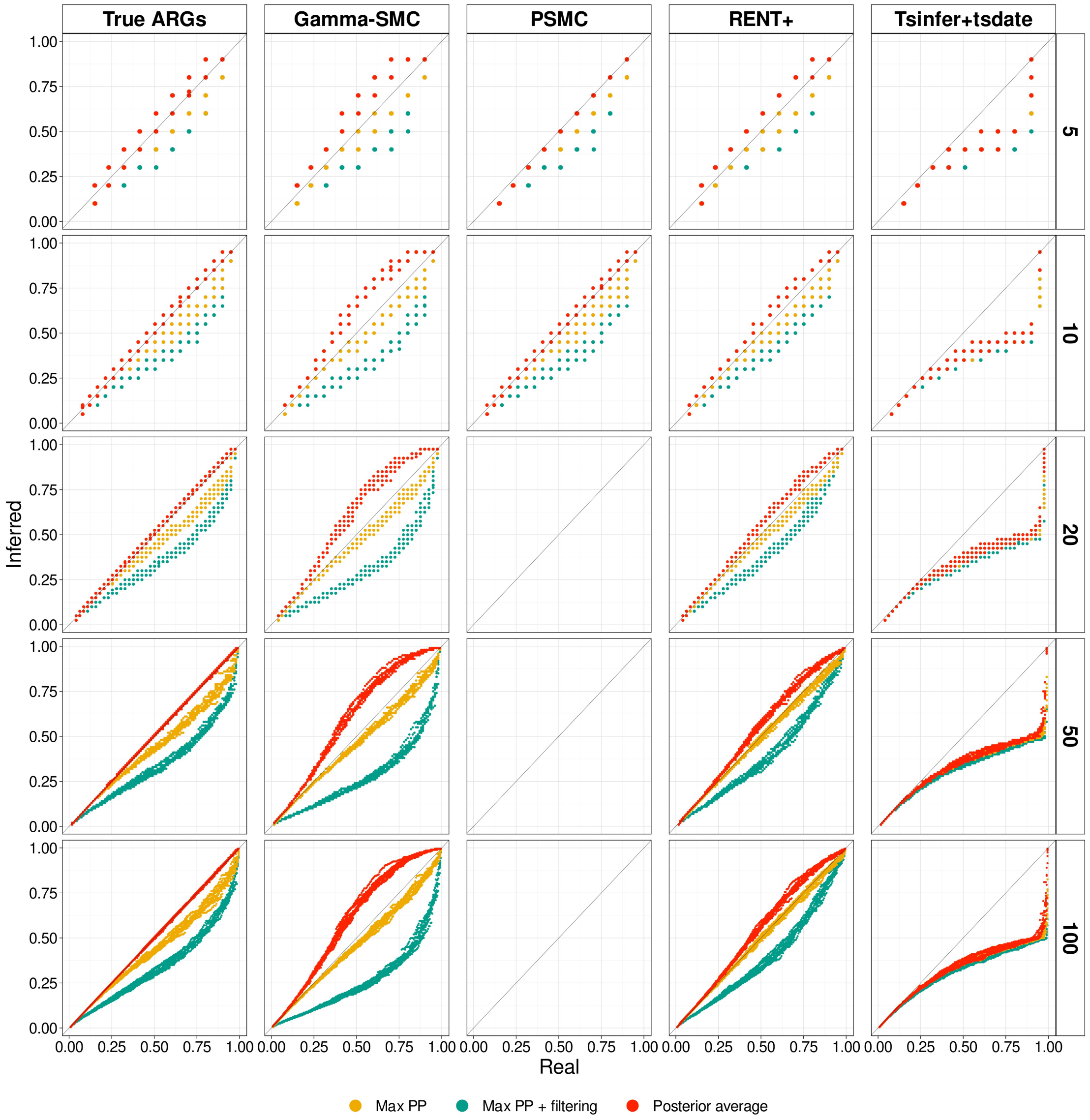

Figure S9. uSFS inference with different ARG reconstruction methods under an increasing population size scenario. The panels show QQ-plots comparing inferred uSFS from different computation approaches (colours) with the true uSFS, using the cumulative proportions from the true uSFS as the reference to obtain corresponding quantiles. Columns correspond to different ARG reconstruction methods and rows correspond to sample sizes.

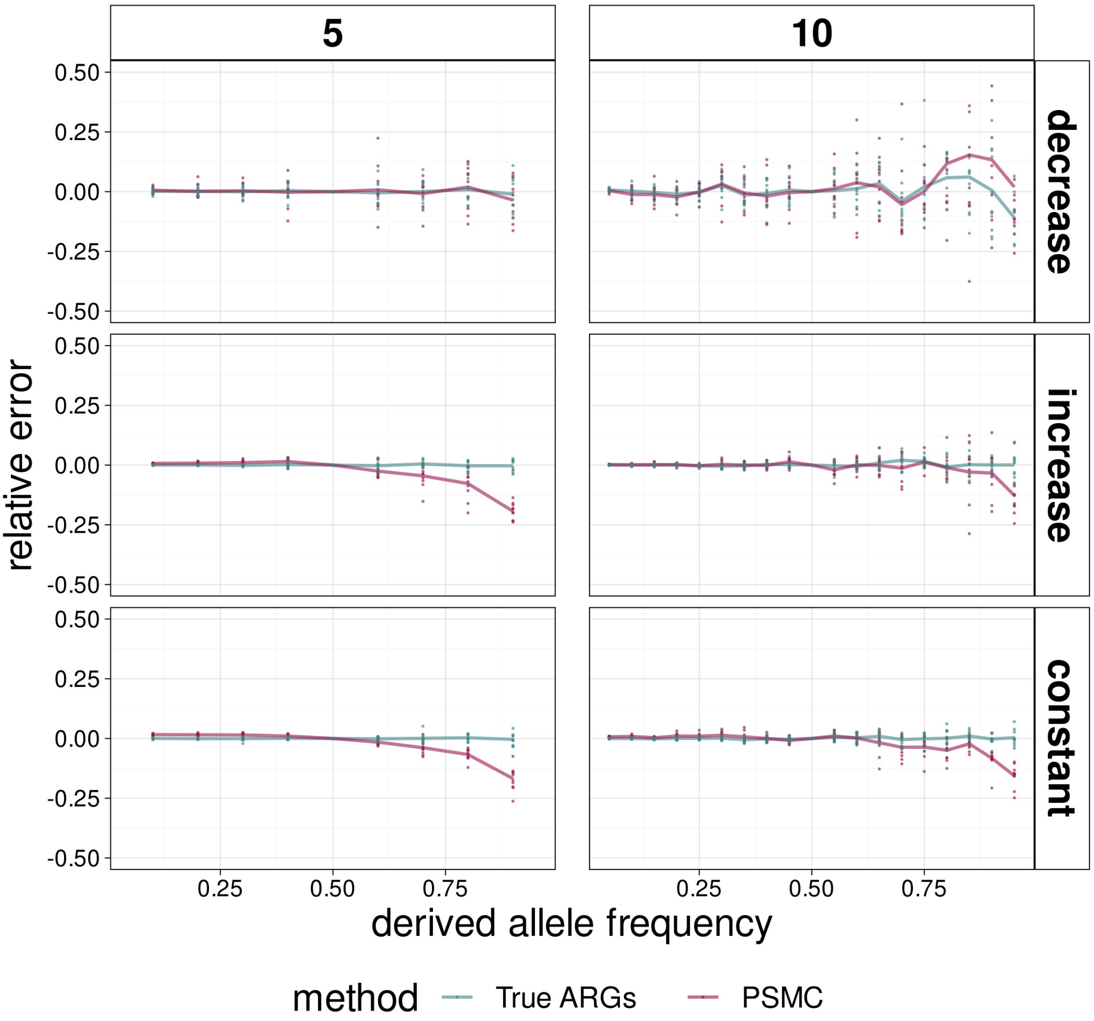

Figure S10. Relative error of uSFS inference from simulations. uSFS estimated by PolarBEAR with true ARGs (blue) and ARGs from PSMC (pink), under different demographic scenarios (rows) and sample sizes columns). The points show 10 replicates in each group, and the lines represent their means. Some of the points with large variability are not shown because of the y-axis limits, especially in decreasing population sizes.

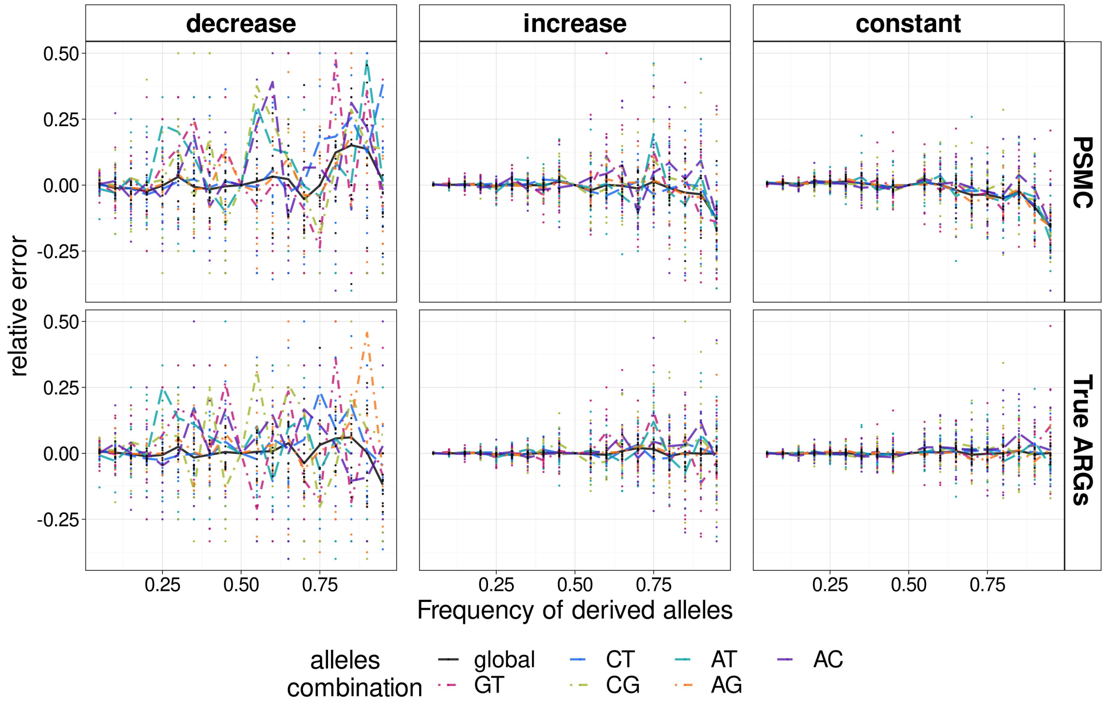

Figure S11. Relative error of uSFS estimated from simulations under distinct transition and transversion rates. colours show different allele combinations. Ancestral alleles estimated by PolarBEAR with true ARGs and ARGs from PSMC (rows), under simulations with different demographic scenarios (columns). Ten replicates were simulated in each case using Kimura's two-parameter model with $\kappa = 5$. The points show 10 replicates in each group, and the lines represent their means. Some of the points with large variability are not shown because of the y-axis limits, especially in decreasing population sizes.

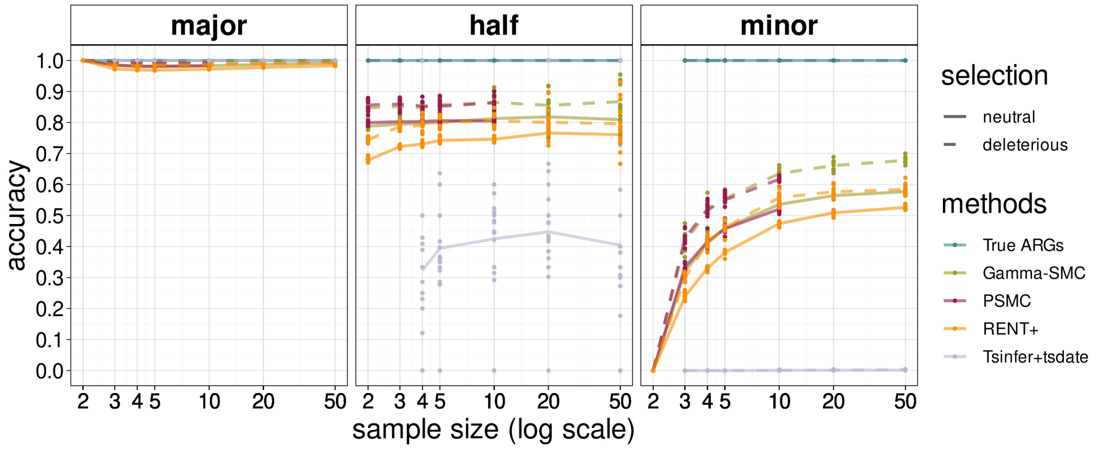

Figure S12. Effects of purifying selection on the Polarisation accuracy of PolarBEAR with ARGs inferred by gamma-SMC, PSMC, RENT+, and tsinfer+tsdate (shown in different colours). Solid lines represent SNPs carrying neutral mutations (including those affected by background selection), and dashed lines represent SNPs carrying deleterious mutations. Panels correspond to accuracy for SNPs classified by their ancestral allele frequency (major, equal, or minor), for different sample sizes. The points show 10 replicates in each group, and the lines represent their means.

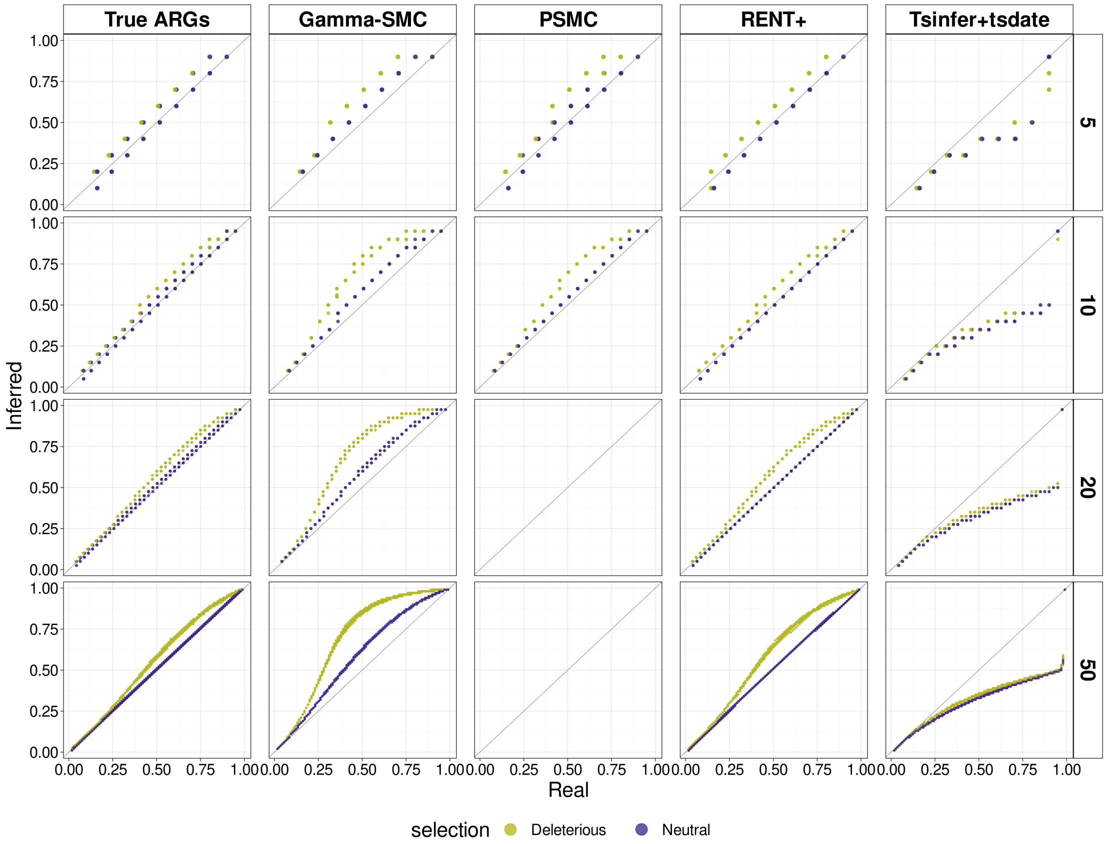

Figure S13. uSFS inference for sites with neutral and deleterious mutations (colours). The panels show QQ-plots comparing inferred uSFS with the true uSFS, using the cumulative proportions from the true uSFS as the reference to obtain corresponding quantiles. Columns correspond to different ARG reconstruction methods and rows correspond to sample sizes.

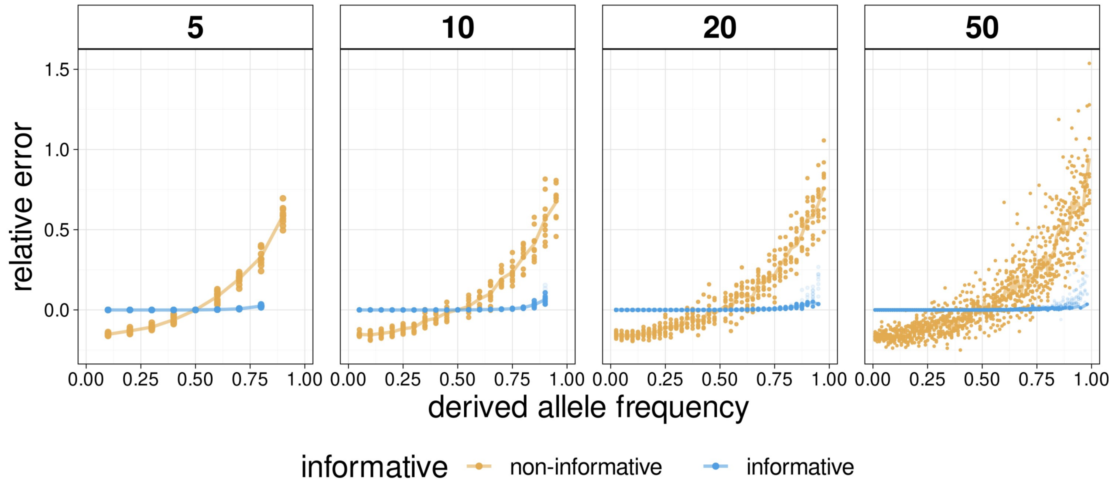

Figure S14. Relative error of uSFS estimated from sites under purifying selection with non-informative or informative genealogies. The points show 10 replicates in each group, and the lines represent their means. The lighter points indicate data where the number of true SNPs does not exceed 10, and are excluded from the calculation of means. Different panels correspond to different sample sizes.

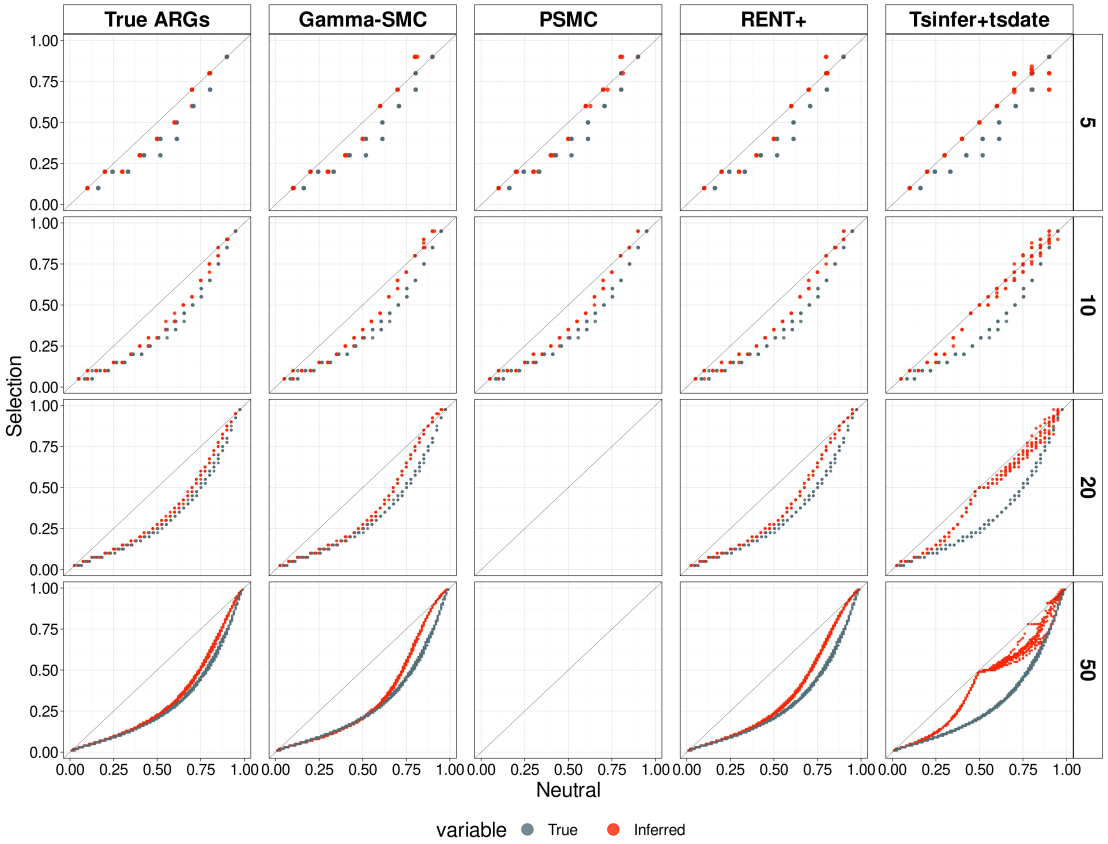

Figure S15. QQ-plots comparing uSFS under neutral and purifying selection scenarios. The cumulative proportions that used to obtain corresponding quantiles are computed from the distributions of uSFS of neutral sites. Distinct colours indicate the true versus inferred uSFS. Columns correspond to different ARG reconstruction methods and rows correspond to sample sizes.

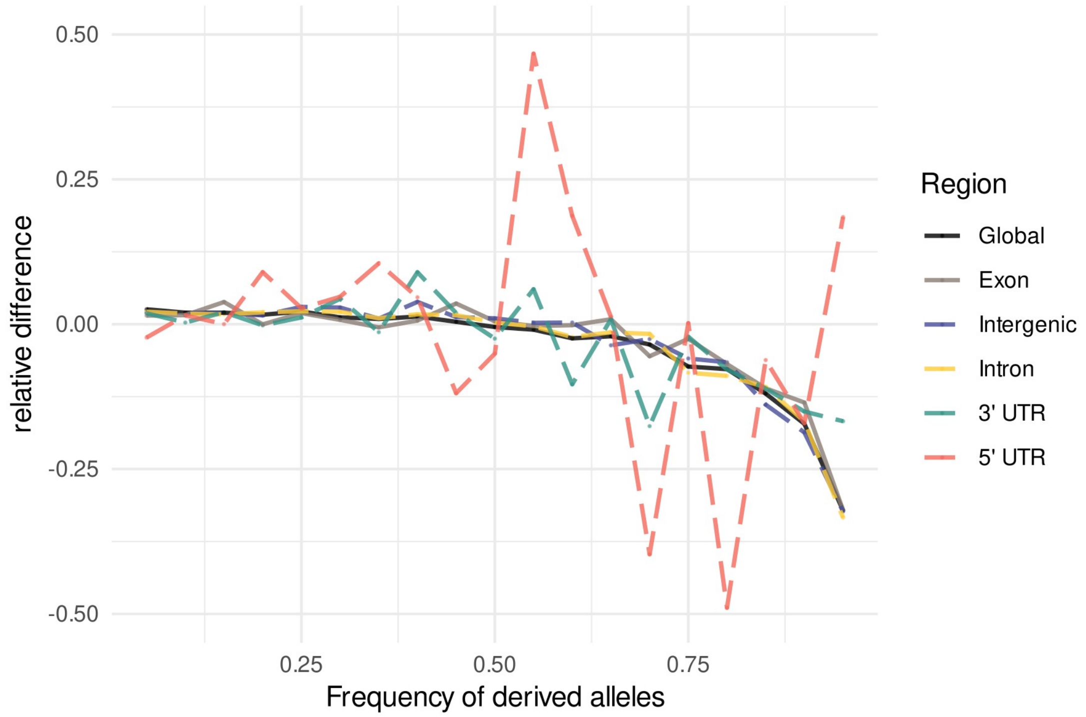

Figure S16. Relative difference between uSFS estimated by PolarBEAR and est-sfs in different genomic regions. The y-axis displays $(PolarBEAR{-}est\_sfs)/_{est\_sfs}$, with SNPs annotated to be in exon, intron, 5’ UTR, 3’UTR and intergenic regions, and the full chromosome (in different colours). Data from human chromosome 1.

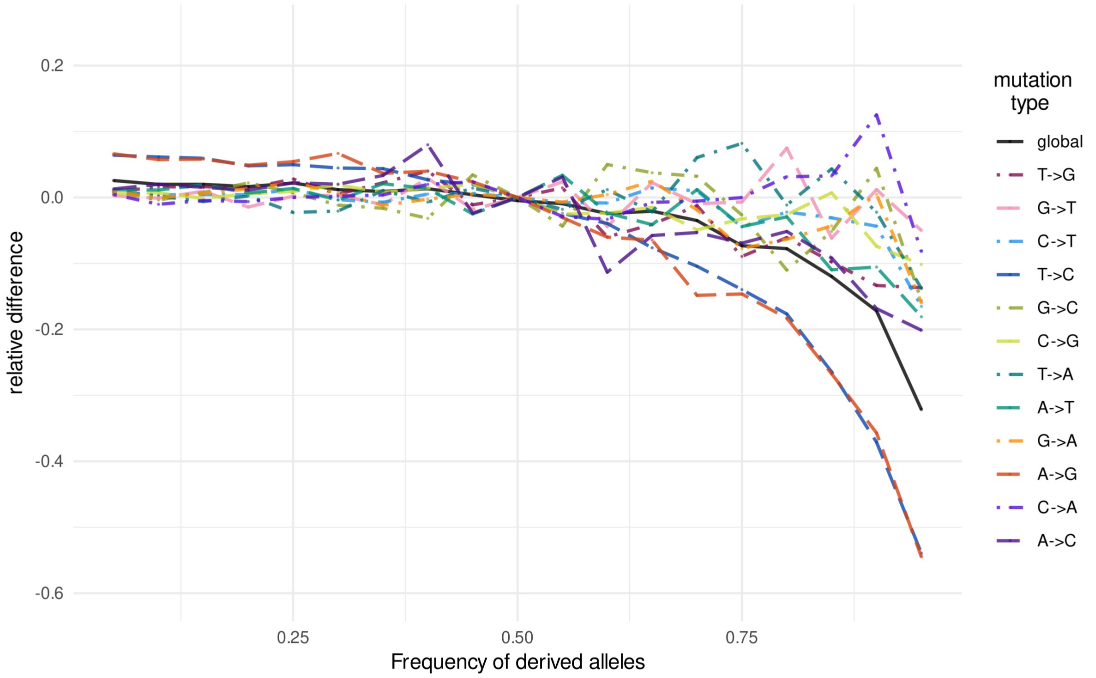

Figure S17. Relative difference between uSFS estimated by PolarBEAR and est-sfs, for distinct mutation types. The y-axis displays $(PolarBEAR - est\_sfs)/_{est\_sfs}$, with SNPs with different mutation types (in different colours) oriented by Polarised states from est-sfs. Data from human chromosome 1.

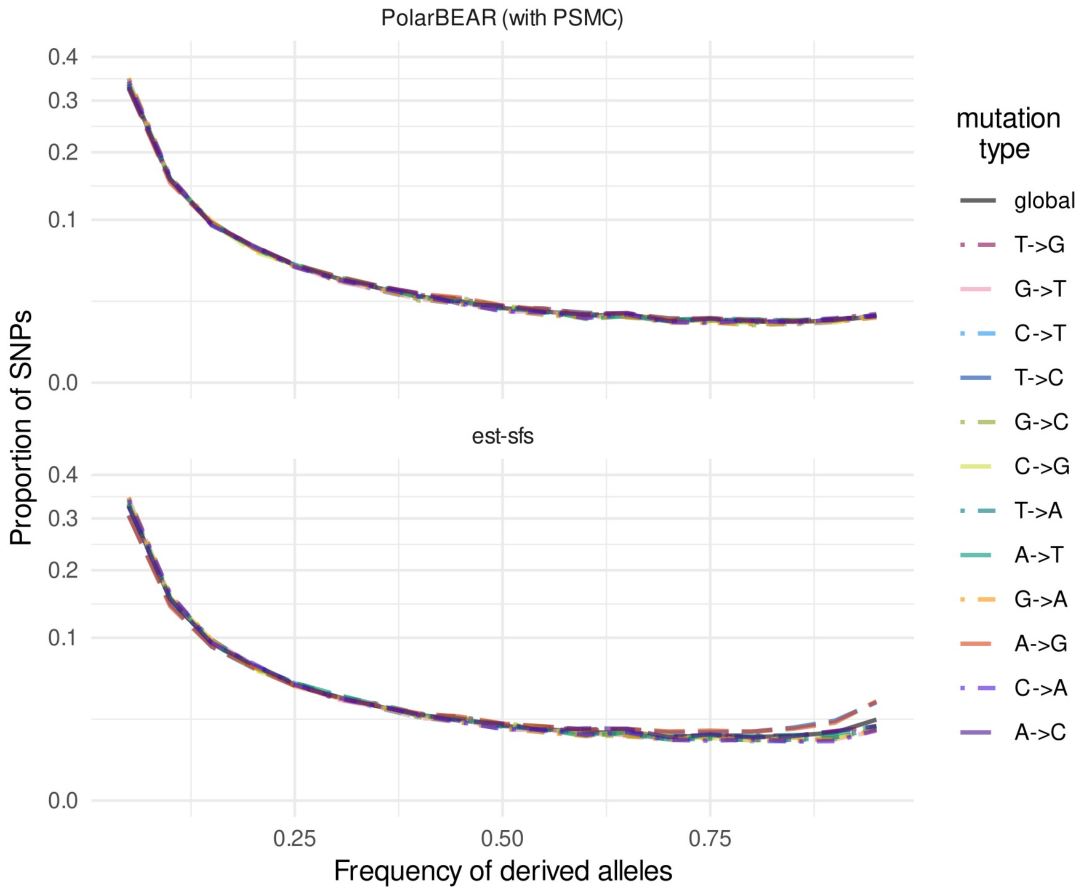

Figure S18. Estimated uSFS for different mutation types. Unfolded SFS estimated by PolarBEAR with PSMC (top) and est-sfs (bottom), SNPs with different mutation types (in different colours) oriented by Polarised states from est-sfs. The y-axis, the proportion of SNPs, is scaled using a square root transformation. Data from human chromosome 1.

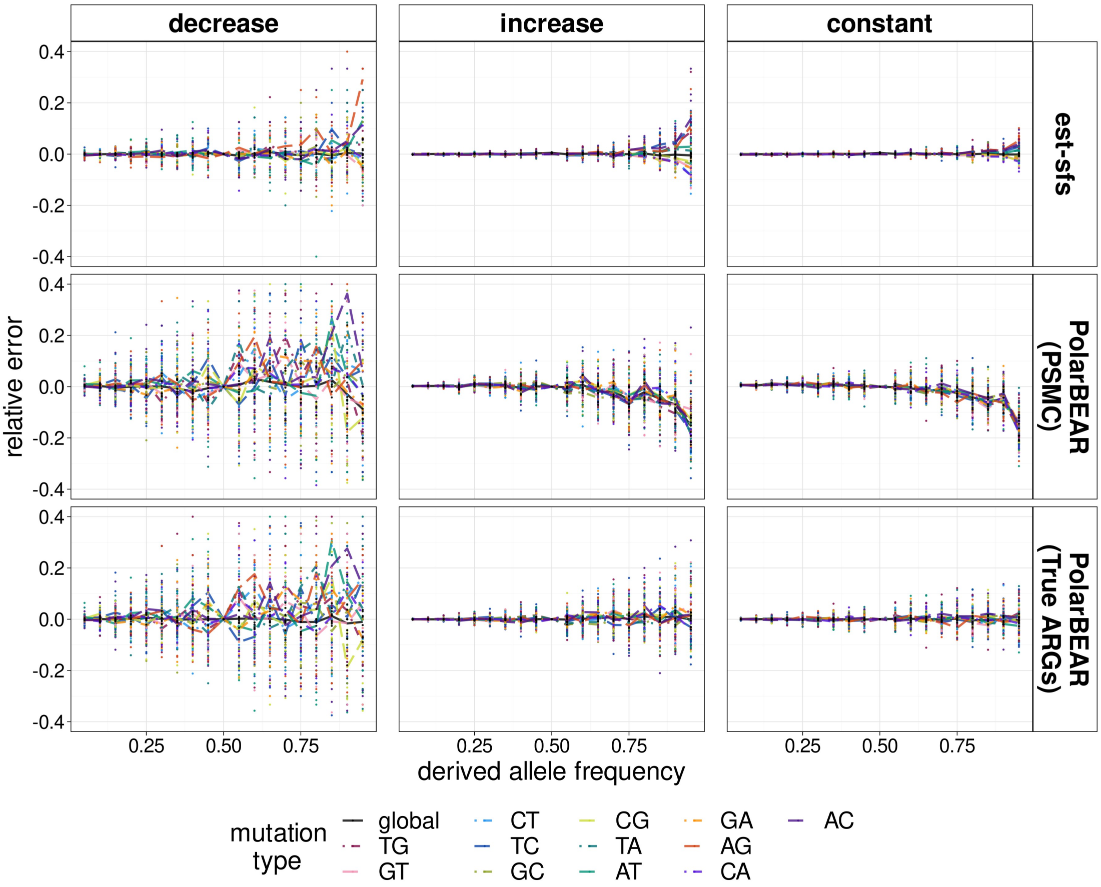

Figure S19. Relative error of uSFS estimated from simulations under unequal GC content. colours show different allele combinations. Ancestral alleles estimated by est-sfs and by PolarBEAR with true ARGs and ARGs from PSMC (rows), under simulations with different demographic scenarios (columns). Ten replicates were simulated in each case using unequal GC content (60%) in the ancestral distribution. The points show 10 replicates in each group, and the lines represent their means. Some of the points with large variability are not shown because of the y-axis limits, especially in decreasing population sizes.

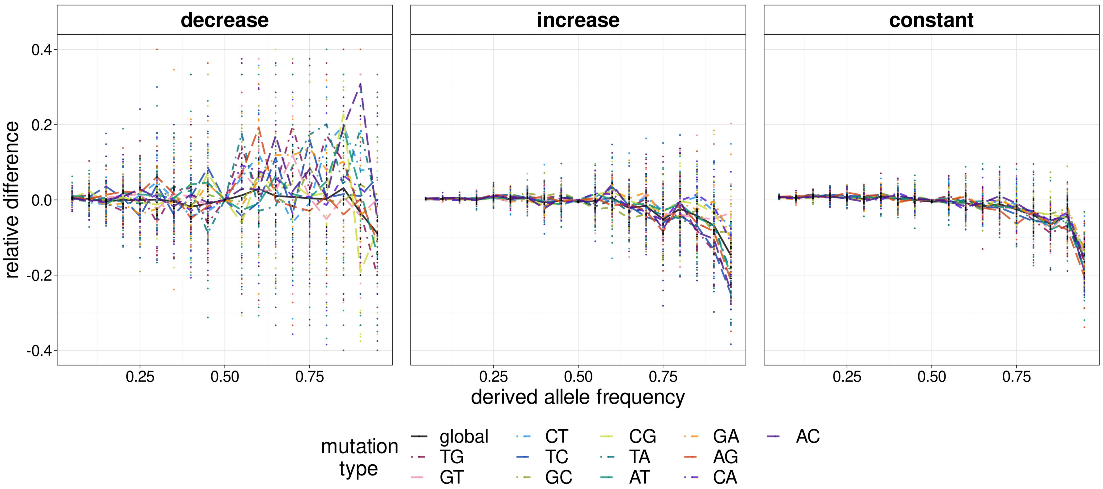

Figure S20. Relative differences between uSFS estimated by PolarBEAR and est-sfs under unequal GC content. The y-axis displays $(PolarBEAR - est\_sfs)/_{est\_sfs}$, with SNPs with different mutation types (in different colours) oriented by Polarised states from est-sfs. Data simulated with unequal GC content (60%) in the ancestral distribution and under distinct demographic scenarios.
